# Supplementary material for: Viewing the US presidential electoral map through the lens of public health
Source: PLoS One. 2021 Jul 21;16(7):e0254001. doi: 10.1371/journal.pone.0254001 (PMC8294501; doi:10.1371/journal.pone.0254001)
Supplement: S1 Table — Correlations for counties from all states, counties from battleground states, and counties from states that flipped from Democratic in 2012 to Republican in 2016 are presented. (DOCX) [file pone.0254001.s001.docx]

**S1 Table.** Pearson correlations between all of the public health-related variables we collected with the percentage of voters in the county that voted for Donald Trump or Hillary Clinton, and the Republican margin shift (from 2012 to 2016). Correlations for counties from all states, counties from battleground states, and counties from states that flipped from Democratic in 2012 to Republican in 2016 are presented.

|  | **All States** | **All States** | **All States** | **Battle States** | **Battle States** | **Battle States** | **Flip States** | | **Flip States** | **Flip States** |  |
| --- | --- | --- | --- | --- | --- | --- | --- | --- | --- | --- | --- |
| **Variable** | % Trump 2016 | % Clinton 2016 | Rep. margin change | % Trump 2016 | % Clinton 2016 | Rep. margin change | | % Trump 2016 | % Clinton 2016 | Rep. margin change | category |
| **Graduation Rate** | 0.35 | -0.34 | 0.13 | 0.31 | -0.33 | 0.1 | | 0.16 | -0.16 | -0.02 | Social, Physical and Economic Environment |
|  | (0.32 to 0.38) | (-0.37 to -0.3) | (0.1 to 0.17) | (0.23 to 0.38) | (-0.4 to -0.26) | (0.02 to 0.18) | | (0.03 to 0.28) | (-0.28 to -0.03) | (-0.15 to 0.11) |  |
| **% Some College** | -0.26 | 0.18 | -0.24 | -0.14 | 0.08 | -0.23 | | -0.59 | 0.56 | -0.57 | Social, Physical and Economic Environment |
|  | (-0.29 to -0.23) | (0.15 to 0.22) | (-0.27 to -0.2) | (-0.22 to -0.06) | (0 to 0.16) | (-0.31 to -0.16) | | (-0.67 to -0.5) | (0.47 to 0.64) | (-0.65 to -0.47) |  |
| **% Children in Poverty** | -0.02 | 0.11 | 0.12 | -0.03 | 0.11 | 0.01 | | 0.15 | -0.11 | 0.52 | Social, Physical and Economic Environment |
|  | (-0.06 to 0.01) | (0.08 to 0.15) | (0.08 to 0.15) | (-0.1 to 0.05) | (0.03 to 0.18) | (-0.07 to 0.09) | | (0.02 to 0.27) | (-0.23 to 0.02) | (0.42 to 0.61) |  |
| **% Single-Parent Households** | -0.37 | 0.45 | 0.02 | -0.42 | 0.48 | -0.06 | | -0.2 | 0.23 | 0.24 | Social, Physical and Economic Environment |
|  | (-0.4 to -0.34) | (0.42 to 0.48) | (-0.01 to 0.06) | (-0.48 to -0.35) | (0.41 to 0.54) | (-0.14 to 0.02) | | (-0.32 to -0.07) | (0.11 to 0.35) | (0.12 to 0.36) |  |
| **Violent Crime Rate** | -0.31 | 0.35 | -0.21 | -0.3 | 0.36 | -0.28 | | -0.26 | 0.29 | -0.07 | Social, Physical and Economic Environment |
|  | (-0.34 to -0.28) | (0.32 to 0.38) | (-0.25 to -0.18) | (-0.37 to -0.22) | (0.29 to 0.43) | (-0.35 to -0.2) | | (-0.38 to -0.14) | (0.16 to 0.4) | (-0.2 to 0.06) |  |
| **Injury Death Rate** | 0.28 | -0.27 | 0.26 | 0.17 | -0.14 | 0.19 | | 0.1 | -0.06 | 0.33 | Social, Physical and Economic Environment |
|  | (0.25 to 0.31) | (-0.3 to -0.24) | (0.23 to 0.3) | (0.09 to 0.25) | (-0.22 to -0.06) | (0.11 to 0.27) | | (-0.02 to 0.23) | (-0.18 to 0.07) | (0.21 to 0.44) |  |
| **% Severe Housing Problems** | -0.57 | 0.59 | -0.34 | -0.57 | 0.61 | -0.35 | | -0.55 | 0.56 | -0.15 | Social, Physical and Economic Environment |
|  | (-0.6 to -0.55) | (0.56 to 0.61) | (-0.37 to -0.31) | (-0.62 to -0.51) | (0.56 to 0.66) | (-0.42 to -0.28) | | (-0.63 to -0.45) | (0.46 to 0.64) | (-0.28 to -0.03) |  |
| **% Disconnected Youth** | 0.24 | -0.19 | 0.23 | 0.3 | -0.25 | 0.24 | | 0.23 | -0.21 | 0.47 | Social, Physical and Economic Environment |
|  | (0.19 to 0.28) | (-0.24 to -0.14) | (0.18 to 0.28) | (0.2 to 0.39) | (-0.35 to -0.15) | (0.14 to 0.34) | | (0.09 to 0.37) | (-0.35 to -0.07) | (0.34 to 0.57) |  |
| **Household Income** | -0.24 | 0.18 | -0.34 | -0.2 | 0.14 | -0.3 | | -0.31 | 0.29 | -0.67 | Social, Physical and Economic Environment |
|  | (-0.27 to -0.21) | (0.14 to 0.21) | (-0.37 to -0.31) | (-0.27 to -0.12) | (0.06 to 0.21) | (-0.37 to -0.23) | | (-0.42 to -0.19) | (0.17 to 0.41) | (-0.74 to -0.6) |  |
| **Firearm Fatalities Rate** | 0.24 | -0.21 | 0.13 | 0.22 | -0.17 | 0.14 | | 0.19 | -0.15 | 0.41 | Social, Physical and Economic Environment |
|  | (0.2 to 0.28) | (-0.25 to -0.17) | (0.09 to 0.17) | (0.13 to 0.31) | (-0.26 to -0.07) | (0.04 to 0.23) | | (0.05 to 0.31) | (-0.28 to -0.02) | (0.29 to 0.52) |  |
| **% Homeowners** | 0.47 | -0.47 | 0.34 | 0.43 | -0.43 | 0.49 | | 0.55 | -0.56 | 0.42 | Social, Physical and Economic Environment |
|  | (0.44 to 0.5) | (-0.5 to -0.45) | (0.31 to 0.37) | (0.36 to 0.49) | (-0.49 to -0.36) | (0.43 to 0.55) | | (0.45 to 0.63) | (-0.64 to -0.47) | (0.31 to 0.52) |  |
| **% Severe Housing Cost Burden** | -0.59 | 0.61 | -0.3 | -0.54 | 0.61 | -0.34 | | -0.48 | 0.49 | -0.2 | Social, Physical and Economic Environment |
|  | (-0.61 to -0.56) | (0.59 to 0.63) | (-0.34 to -0.27) | (-0.59 to -0.48) | (0.55 to 0.65) | (-0.41 to -0.27) | | (-0.57 to -0.37) | (0.39 to 0.59) | (-0.32 to -0.08) |  |
| **Asbestosis** | 0.02 | -0.02 | -0.05 | -0.09 | 0.11 | 0.01 | | -0.06 | 0.04 | 0.11 | Respiratory diseases |
|  | (-0.01 to 0.06) | (-0.05 to 0.02) | (-0.09 to -0.02) | (-0.17 to -0.02) | (0.03 to 0.19) | (-0.07 to 0.09) | | (-0.19 to 0.07) | (-0.09 to 0.16) | (-0.02 to 0.23) |  |
| **Asthma** | -0.21 | 0.26 | -0.05 | 0.03 | -0.01 | 0 | | -0.23 | 0.24 | 0.18 | Respiratory diseases |
|  | (-0.25 to -0.18) | (0.23 to 0.29) | (-0.08 to -0.01) | (-0.05 to 0.11) | (-0.09 to 0.07) | (-0.08 to 0.08) | | (-0.34 to -0.1) | (0.11 to 0.35) | (0.06 to 0.3) |  |
| **Chronic obstructive pulmonary** | 0.46 | -0.42 | 0.26 | 0.34 | -0.3 | 0.12 | | 0.17 | -0.19 | 0.47 | Respiratory diseases |
|  | (0.43 to 0.49) | (-0.45 to -0.39) | (0.22 to 0.29) | (0.26 to 0.4) | (-0.37 to -0.23) | (0.04 to 0.2) | | (0.05 to 0.29) | (-0.31 to -0.06) | (0.37 to 0.57) |  |
| **Chronic respiratory diseases** | 0.43 | -0.39 | 0.25 | 0.3 | -0.26 | 0.11 | | 0.14 | -0.15 | 0.46 | Respiratory diseases |
|  | (0.4 to 0.46) | (-0.42 to -0.36) | (0.21 to 0.28) | (0.22 to 0.37) | (-0.34 to -0.19) | (0.03 to 0.18) | | (0.01 to 0.26) | (-0.27 to -0.03) | (0.35 to 0.55) |  |
| **Coal workers pneumoconiosis** | 0.1 | -0.09 | 0.07 | 0.09 | -0.06 | 0.12 | | 0.22 | -0.19 | 0.17 | Respiratory diseases |
|  | (0.06 to 0.13) | (-0.13 to -0.06) | (0.03 to 0.1) | (0.01 to 0.17) | (-0.14 to 0.02) | (0.04 to 0.19) | | (0.1 to 0.34) | (-0.31 to -0.07) | (0.04 to 0.29) |  |
| **Interstitial lung disease** | -0.26 | 0.26 | -0.07 | -0.24 | 0.26 | -0.1 | | -0.3 | 0.27 | -0.07 | Respiratory diseases |
|  | (-0.3 to -0.23) | (0.22 to 0.29) | (-0.1 to -0.03) | (-0.32 to -0.17) | (0.19 to 0.34) | (-0.18 to -0.03) | | (-0.41 to -0.18) | (0.14 to 0.38) | (-0.2 to 0.06) |  |
| **Other chronic respiratory** | 0.11 | -0.06 | 0.07 | 0.11 | -0.08 | 0.1 | | -0.11 | 0.09 | 0.4 | Respiratory diseases |
|  | (0.07 to 0.14) | (-0.1 to -0.03) | (0.04 to 0.11) | (0.03 to 0.19) | (-0.16 to 0) | (0.02 to 0.18) | | (-0.23 to 0.02) | (-0.04 to 0.21) | (0.29 to 0.5) |  |
| **Other pneumoconiosis** | 0.25 | -0.18 | 0.01 | 0.3 | -0.29 | 0.27 | | 0.54 | -0.51 | 0.34 | Respiratory diseases |
|  | (0.21 to 0.28) | (-0.22 to -0.15) | (-0.03 to 0.04) | (0.23 to 0.37) | (-0.36 to -0.22) | (0.19 to 0.34) | | (0.44 to 0.62) | (-0.6 to -0.41) | (0.22 to 0.45) |  |
| **Pneumoconiosis** | 0.11 | -0.1 | 0.05 | 0.05 | -0.04 | 0.13 | | 0.22 | -0.2 | 0.22 | Respiratory diseases |
|  | (0.08 to 0.15) | (-0.13 to -0.07) | (0.02 to 0.09) | (-0.03 to 0.13) | (-0.12 to 0.04) | (0.05 to 0.21) | | (0.1 to 0.34) | (-0.32 to -0.07) | (0.09 to 0.33) |  |
| **Silicosis** | -0.01 | -0.01 | -0.02 | -0.04 | -0.05 | 0.01 | | 0.2 | -0.2 | 0.05 | Respiratory diseases |
|  | (-0.05 to 0.02) | (-0.05 to 0.02) | (-0.06 to 0.01) | (-0.12 to 0.04) | (-0.13 to 0.03) | (-0.06 to 0.09) | | (0.08 to 0.32) | (-0.32 to -0.08) | (-0.08 to 0.18) |  |
| **Mortality risk, age 0-5** | 0.07 | 0.02 | 0.01 | -0.08 | 0.16 | -0.17 | | -0.1 | 0.15 | 0.26 | Life expectancy and Mortality |
|  | (0.04 to 0.11) | (-0.02 to 0.05) | (-0.03 to 0.05) | (-0.16 to 0) | (0.08 to 0.24) | (-0.25 to -0.09) | | (-0.22 to 0.03) | (0.02 to 0.27) | (0.14 to 0.38) |  |
| **Mortality risk, age 25-45** | 0.11 | -0.02 | 0.08 | -0.02 | 0.09 | -0.05 | | 0.06 | 0 | 0.47 | Life expectancy and Mortality |
|  | (0.07 to 0.14) | (-0.06 to 0.01) | (0.04 to 0.11) | (-0.1 to 0.06) | (0.01 to 0.17) | (-0.13 to 0.03) | | (-0.07 to 0.18) | (-0.13 to 0.13) | (0.37 to 0.57) |  |
| **Mortality risk, age 45-65** | 0.16 | -0.06 | 0.15 | 0.01 | 0.08 | 0.01 | | 0.07 | -0.02 | 0.47 | Life expectancy and Mortality |
|  | (0.13 to 0.19) | (-0.1 to -0.03) | (0.11 to 0.18) | (-0.07 to 0.08) | (0 to 0.15) | (-0.07 to 0.09) | | (-0.06 to 0.19) | (-0.14 to 0.11) | (0.36 to 0.56) |  |
| **Mortality risk, age 5-25** | 0.21 | -0.13 | 0.11 | 0.1 | -0.04 | 0.03 | | 0.26 | -0.22 | 0.58 | Life expectancy and Mortality |
|  | (0.18 to 0.25) | (-0.17 to -0.1) | (0.07 to 0.14) | (0.02 to 0.18) | (-0.12 to 0.04) | (-0.05 to 0.11) | | (0.14 to 0.38) | (-0.34 to -0.1) | (0.49 to 0.66) |  |
| **Mortality risk, age 65-85** | 0.25 | -0.17 | 0.2 | -0.02 | 0.07 | 0.12 | | 0.16 | -0.13 | 0.43 | Life expectancy and Mortality |
|  | (0.21 to 0.28) | (-0.2 to -0.13) | (0.16 to 0.23) | (-0.1 to 0.06) | (-0.01 to 0.15) | (0.04 to 0.2) | | (0.03 to 0.28) | (-0.26 to -0.01) | (0.32 to 0.53) |  |
| **prct_male_under_18_medicaid** | -0.1 | 0.17 | 0.14 | -0.15 | 0.22 | 0.08 | | 0.11 | -0.1 | 0.5 | Insurance and Healthcare cost |
|  | (-0.14 to -0.07) | (0.14 to 0.21) | (0.1 to 0.17) | (-0.22 to -0.07) | (0.14 to 0.29) | (0 to 0.15) | | (-0.02 to 0.23) | (-0.22 to 0.03) | (0.39 to 0.59) |  |
| **prct_male_18_64_medicaid** | -0.16 | 0.17 | 0.29 | -0.24 | 0.23 | 0.34 | | 0.1 | -0.1 | 0.56 | Insurance and Healthcare cost |
|  | (-0.19 to -0.12) | (0.14 to 0.21) | (0.26 to 0.32) | (-0.31 to -0.16) | (0.16 to 0.31) | (0.27 to 0.41) | | (-0.03 to 0.22) | (-0.22 to 0.03) | (0.47 to 0.65) |  |
| **prct_male_over_64_medicaid** | -0.19 | 0.25 | 0 | -0.19 | 0.25 | 0.07 | | -0.15 | 0.15 | 0.32 | Insurance and Healthcare cost |
|  | (-0.22 to -0.16) | (0.22 to 0.28) | (-0.03 to 0.04) | (-0.27 to -0.12) | (0.17 to 0.32) | (-0.01 to 0.15) | | (-0.28 to -0.03) | (0.02 to 0.27) | (0.2 to 0.43) |  |
| **prct_male_medicaid** | -0.16 | 0.21 | 0.19 | -0.25 | 0.28 | 0.2 | | 0.03 | -0.03 | 0.53 | Insurance and Healthcare cost |
|  | (-0.19 to -0.12) | (0.18 to 0.24) | (0.16 to 0.22) | (-0.32 to -0.17) | (0.21 to 0.35) | (0.13 to 0.28) | | (-0.09 to 0.16) | (-0.16 to 0.1) | (0.43 to 0.61) |  |
| **prct_female_under_18_medicaid** | -0.1 | 0.17 | 0.14 | -0.12 | 0.2 | 0.09 | | 0.12 | -0.11 | 0.49 | Insurance and Healthcare cost |
|  | (-0.13 to -0.06) | (0.13 to 0.2) | (0.11 to 0.18) | (-0.2 to -0.05) | (0.12 to 0.27) | (0.01 to 0.16) | | (0 to 0.25) | (-0.23 to 0.02) | (0.38 to 0.58) |  |
| **prct_female_18_64_medicaid** | -0.16 | 0.19 | 0.31 | -0.24 | 0.24 | 0.35 | | 0.09 | -0.08 | 0.55 | Insurance and Healthcare cost |
|  | (-0.2 to -0.13) | (0.15 to 0.22) | (0.28 to 0.34) | (-0.32 to -0.17) | (0.16 to 0.31) | (0.28 to 0.42) | | (-0.04 to 0.21) | (-0.2 to 0.05) | (0.45 to 0.63) |  |
| **prct_female_over_64_medicaid** | -0.21 | 0.28 | 0 | -0.26 | 0.31 | -0.01 | | -0.14 | 0.14 | 0.31 | Insurance and Healthcare cost |
|  | (-0.24 to -0.17) | (0.25 to 0.31) | (-0.03 to 0.04) | (-0.33 to -0.18) | (0.24 to 0.38) | (-0.09 to 0.07) | | (-0.27 to -0.02) | (0.01 to 0.26) | (0.19 to 0.42) |  |
| **prct_female_medicaid** | -0.15 | 0.21 | 0.22 | -0.23 | 0.27 | 0.23 | | 0.04 | -0.04 | 0.53 | Insurance and Healthcare cost |
|  | (-0.19 to -0.12) | (0.18 to 0.24) | (0.18 to 0.25) | (-0.31 to -0.16) | (0.19 to 0.34) | (0.15 to 0.3) | | (-0.09 to 0.17) | (-0.16 to 0.09) | (0.43 to 0.61) |  |
| **prcnt_no_highs_25_64_with_ins** | -0.05 | 0.02 | 0.32 | -0.02 | -0.05 | 0.31 | | -0.02 | 0.01 | 0.08 | Insurance and Healthcare cost |
|  | (-0.08 to -0.01) | (-0.01 to 0.06) | (0.29 to 0.35) | (-0.09 to 0.06) | (-0.13 to 0.03) | (0.24 to 0.38) | | (-0.14 to 0.11) | (-0.11 to 0.14) | (-0.05 to 0.2) |  |
| **prcnt_no_highs_25_64_with_private_ins** | 0.12 | -0.16 | -0.02 | 0.25 | -0.29 | 0.04 | | 0.1 | -0.1 | -0.23 | Insurance and Healthcare cost |
|  | (0.08 to 0.15) | (-0.19 to -0.13) | (-0.05 to 0.02) | (0.17 to 0.32) | (-0.37 to -0.22) | (-0.04 to 0.12) | | (-0.03 to 0.22) | (-0.23 to 0.02) | (-0.35 to -0.11) |  |
| **prcnt_no_highs_25_64_with_public_ins** | -0.16 | 0.17 | 0.33 | -0.27 | 0.25 | 0.29 | | -0.08 | 0.08 | 0.29 | Insurance and Healthcare cost |
|  | (-0.19 to -0.12) | (0.14 to 0.21) | (0.3 to 0.36) | (-0.34 to -0.2) | (0.17 to 0.32) | (0.22 to 0.36) | | (-0.21 to 0.05) | (-0.04 to 0.21) | (0.17 to 0.41) |  |
| **prcnt_yes_highs_25_64_with_ins** | 0.01 | -0.05 | 0.32 | 0.03 | -0.11 | 0.4 | | 0.2 | -0.2 | -0.08 | Insurance and Healthcare cost |
|  | (-0.03 to 0.04) | (-0.09 to -0.02) | (0.29 to 0.35) | (-0.05 to 0.11) | (-0.18 to -0.03) | (0.33 to 0.46) | | (0.08 to 0.32) | (-0.32 to -0.08) | (-0.2 to 0.05) |  |
| **prcnt_yes_highs_25_64_with_private_ins** | 0.18 | -0.23 | 0.1 | 0.26 | -0.31 | 0.15 | | 0.21 | -0.2 | -0.26 | Insurance and Healthcare cost |
|  | (0.15 to 0.22) | (-0.26 to -0.19) | (0.07 to 0.14) | (0.19 to 0.34) | (-0.38 to -0.24) | (0.07 to 0.23) | | (0.08 to 0.32) | (-0.32 to -0.08) | (-0.38 to -0.14) |  |
| **prcnt_yes_highs_25_64_with_public_ins** | -0.23 | 0.25 | 0.22 | -0.34 | 0.34 | 0.26 | | -0.1 | 0.09 | 0.38 | Insurance and Healthcare cost |
|  | (-0.27 to -0.2) | (0.21 to 0.28) | (0.18 to 0.25) | (-0.41 to -0.27) | (0.27 to 0.41) | (0.18 to 0.33) | | (-0.22 to 0.03) | (-0.03 to 0.22) | (0.27 to 0.48) |  |
| **prcnt_bachelor_25_64_with_ins** | 0.02 | -0.05 | 0.09 | 0.05 | -0.12 | 0.17 | | -0.06 | 0.07 | -0.25 | Insurance and Healthcare cost |
|  | (-0.01 to 0.06) | (-0.08 to -0.01) | (0.06 to 0.13) | (-0.03 to 0.13) | (-0.19 to -0.04) | (0.09 to 0.25) | | (-0.19 to 0.07) | (-0.06 to 0.19) | (-0.37 to -0.13) |  |
| **prcnt_bachelor_25_64_with_private_ins** | 0.1 | -0.11 | -0.04 | 0.11 | -0.15 | -0.05 | | -0.13 | 0.13 | -0.41 | Insurance and Healthcare cost |
|  | (0.06 to 0.13) | (-0.15 to -0.08) | (-0.08 to -0.01) | (0.03 to 0.19) | (-0.23 to -0.07) | (-0.13 to 0.03) | | (-0.25 to 0) | (0.01 to 0.26) | (-0.51 to -0.29) |  |
| **prcnt_bachelor_25_64_with_public_ins** | -0.11 | 0.11 | 0.19 | -0.1 | 0.1 | 0.27 | | 0.21 | -0.22 | 0.47 | Insurance and Healthcare cost |
|  | (-0.15 to -0.08) | (0.08 to 0.15) | (0.15 to 0.22) | (-0.17 to -0.02) | (0.02 to 0.18) | (0.19 to 0.34) | | (0.09 to 0.33) | (-0.34 to -0.1) | (0.36 to 0.56) |  |
| **wnh_prcnt_18_dis** | 0.1 | -0.08 | 0.14 | 0.02 | 0.02 | 0.17 | | 0.32 | -0.32 | 0.31 | Insurance and Healthcare cost |
|  | (0.07 to 0.14) | (-0.12 to -0.05) | (0.11 to 0.18) | (-0.05 to 0.1) | (-0.06 to 0.1) | (0.09 to 0.24) | | (0.2 to 0.43) | (-0.43 to -0.2) | (0.19 to 0.42) |  |
| **wnh_prcnt_18_64_dis** | 0.26 | -0.21 | 0.26 | 0.09 | -0.04 | 0.21 | | 0.38 | -0.37 | 0.62 | Insurance and Healthcare cost |
|  | (0.22 to 0.29) | (-0.24 to -0.17) | (0.22 to 0.29) | (0.01 to 0.17) | (-0.11 to 0.04) | (0.13 to 0.28) | | (0.26 to 0.48) | (-0.48 to -0.26) | (0.53 to 0.69) |  |
| **wnh_prcnt_65_dis** | 0.3 | -0.25 | 0.15 | 0.22 | -0.17 | 0.09 | | 0.34 | -0.32 | 0.44 | Insurance and Healthcare cost |
|  | (0.27 to 0.34) | (-0.29 to -0.22) | (0.11 to 0.18) | (0.14 to 0.29) | (-0.25 to -0.09) | (0.01 to 0.17) | | (0.22 to 0.45) | (-0.43 to -0.2) | (0.33 to 0.54) |  |
| **w_prcnt_18_dis** | 0.09 | -0.06 | 0.16 | 0.03 | 0 | 0.18 | | 0.31 | -0.3 | 0.3 | Insurance and Healthcare cost |
|  | (0.05 to 0.12) | (-0.1 to -0.03) | (0.13 to 0.2) | (-0.05 to 0.11) | (-0.08 to 0.08) | (0.1 to 0.26) | | (0.19 to 0.42) | (-0.41 to -0.18) | (0.18 to 0.41) |  |
| **w_prcnt_18_64_dis** | 0.26 | -0.21 | 0.29 | 0.1 | -0.05 | 0.24 | | 0.38 | -0.37 | 0.61 | Insurance and Healthcare cost |
|  | (0.23 to 0.3) | (-0.24 to -0.18) | (0.26 to 0.32) | (0.02 to 0.18) | (-0.13 to 0.03) | (0.16 to 0.31) | | (0.27 to 0.48) | (-0.48 to -0.26) | (0.53 to 0.69) |  |
| **w_prcnt_65_dis** | 0.27 | -0.22 | 0.12 | 0.2 | -0.16 | 0.07 | | 0.34 | -0.31 | 0.44 | Insurance and Healthcare cost |
|  | (0.24 to 0.3) | (-0.25 to -0.19) | (0.09 to 0.16) | (0.12 to 0.28) | (-0.24 to -0.09) | (-0.01 to 0.15) | | (0.22 to 0.44) | (-0.42 to -0.19) | (0.33 to 0.53) |  |
| **Uninsured %: <= 138% of Poverty** | 0.25 | -0.2 | -0.37 | 0.17 | -0.1 | -0.44 | | -0.13 | 0.11 | -0.12 | Insurance and Healthcare cost |
|  | (0.22 to 0.28) | (-0.24 to -0.17) | (-0.4 to -0.34) | (0.09 to 0.25) | (-0.18 to -0.02) | (-0.5 to -0.37) | | (-0.26 to -0.01) | (-0.02 to 0.23) | (-0.24 to 0.01) |  |
| **Uninsured %: <= 400% of Poverty** | 0.19 | -0.13 | -0.34 | 0.07 | 0 | -0.41 | | -0.13 | 0.12 | 0.04 | Insurance and Healthcare cost |
|  | (0.15 to 0.22) | (-0.17 to -0.1) | (-0.37 to -0.31) | (-0.01 to 0.15) | (-0.08 to 0.08) | (-0.47 to -0.34) | | (-0.26 to -0.01) | (-0.01 to 0.24) | (-0.09 to 0.16) |  |
| **Uninsured %: All Incomes** | 0.22 | -0.15 | -0.24 | 0.12 | -0.04 | -0.29 | | 0.02 | -0.03 | 0.31 | Insurance and Healthcare cost |
|  | (0.18 to 0.25) | (-0.19 to -0.12) | (-0.27 to -0.2) | (0.04 to 0.2) | (-0.12 to 0.04) | (-0.36 to -0.22) | | (-0.11 to 0.14) | (-0.16 to 0.1) | (0.19 to 0.42) |  |
| **Part B Drugs Actual Costs** | -0.15 | 0.02 | -0.16 | -0.29 | 0.32 | -0.37 | | -0.44 | 0.48 | -0.47 | Insurance and Healthcare cost |
|  | (-0.19 to -0.12) | (-0.01 to 0.06) | (-0.2 to -0.13) | (-0.36 to -0.21) | (0.25 to 0.39) | (-0.44 to -0.3) | | (-0.54 to -0.33) | (0.38 to 0.57) | (-0.56 to -0.36) |  |
| **Emergency Department Visits** | -0.15 | 0.02 | -0.16 | -0.41 | 0.44 | -0.39 | | -0.49 | 0.52 | -0.39 | Insurance and Healthcare cost |
|  | (-0.19 to -0.12) | (-0.01 to 0.06) | (-0.2 to -0.13) | (-0.47 to -0.34) | (0.38 to 0.5) | (-0.46 to -0.32) | | (-0.58 to -0.38) | (0.42 to 0.61) | (-0.49 to -0.27) |  |
| **Imaging Per Capita Actual Costs** | -0.17 | 0.22 | -0.26 | -0.13 | 0.19 | -0.39 | | -0.05 | 0.1 | -0.21 | Insurance and Healthcare cost |
|  | (-0.2 to -0.13) | (0.18 to 0.25) | (-0.29 to -0.23) | (-0.21 to -0.05) | (0.12 to 0.27) | (-0.45 to -0.32) | | (-0.18 to 0.08) | (-0.03 to 0.22) | (-0.33 to -0.09) |  |
| **Procedures Per Capita Actual Costs** | -0.22 | 0.25 | -0.4 | -0.08 | 0.15 | -0.46 | | -0.02 | 0.08 | -0.34 | Insurance and Healthcare cost |
|  | (-0.25 to -0.19) | (0.21 to 0.28) | (-0.43 to -0.37) | (-0.15 to 0) | (0.07 to 0.22) | (-0.52 to -0.39) | | (-0.14 to 0.11) | (-0.05 to 0.2) | (-0.44 to -0.22) |  |
| **Hospice Per Capita Actual Costs** | -0.02 | 0.06 | -0.22 | -0.14 | 0.17 | -0.35 | | -0.2 | 0.18 | -0.32 | Insurance and Healthcare cost |
|  | (-0.06 to 0.01) | (0.02 to 0.09) | (-0.25 to -0.19) | (-0.22 to -0.06) | (0.09 to 0.25) | (-0.42 to -0.28) | | (-0.32 to -0.07) | (0.06 to 0.31) | (-0.43 to -0.21) |  |
| **Tests Per Capita Actual Costs** | -0.1 | 0.17 | -0.26 | -0.13 | 0.22 | -0.45 | | -0.14 | 0.19 | -0.25 | Insurance and Healthcare cost |
|  | (-0.14 to -0.07) | (0.14 to 0.2) | (-0.29 to -0.22) | (-0.21 to -0.05) | (0.14 to 0.29) | (-0.51 to -0.38) | | (-0.26 to -0.01) | (0.06 to 0.31) | (-0.37 to -0.13) |  |
| **Actual Per Capita Costs** | -0.07 | 0.13 | -0.1 | 0 | 0.05 | -0.1 | | -0.1 | 0.16 | -0.16 | Insurance and Healthcare cost |
|  | (-0.1 to -0.03) | (0.1 to 0.17) | (-0.13 to -0.06) | (-0.08 to 0.08) | (-0.03 to 0.12) | (-0.18 to -0.02) | | (-0.23 to 0.02) | (0.04 to 0.28) | (-0.28 to -0.03) |  |
| **Percent Eligible for Medicaid** | -0.14 | 0.24 | 0.15 | -0.31 | 0.34 | 0.21 | | -0.13 | 0.11 | 0.34 | Insurance and Healthcare cost |
|  | (-0.17 to -0.1) | (0.21 to 0.27) | (0.12 to 0.19) | (-0.38 to -0.23) | (0.27 to 0.41) | (0.13 to 0.28) | | (-0.25 to 0) | (-0.01 to 0.24) | (0.22 to 0.44) |  |
| **Percent Male** | 0.18 | -0.21 | 0.18 | 0.13 | -0.16 | 0.39 | | 0.32 | -0.33 | 0.52 | Insurance and Healthcare cost |
|  | (0.15 to 0.22) | (-0.24 to -0.17) | (0.15 to 0.22) | (0.05 to 0.2) | (-0.24 to -0.08) | (0.32 to 0.46) | | (0.2 to 0.43) | (-0.44 to -0.21) | (0.42 to 0.61) |  |
| **Percent Female** | -0.18 | 0.21 | -0.18 | -0.13 | 0.16 | -0.39 | | -0.32 | 0.33 | -0.52 | Insurance and Healthcare cost |
|  | (-0.22 to -0.15) | (0.17 to 0.24) | (-0.22 to -0.15) | (-0.2 to -0.05) | (0.08 to 0.24) | (-0.46 to -0.32) | | (-0.43 to -0.2) | (0.21 to 0.44) | (-0.61 to -0.42) |  |
| **Diarrheal diseases** | -0.11 | 0.14 | 0.12 | -0.23 | 0.27 | -0.27 | | 0.12 | -0.1 | -0.22 | Infectious diseases |
|  | (-0.14 to -0.08) | (0.1 to 0.17) | (0.08 to 0.15) | (-0.3 to -0.15) | (0.2 to 0.34) | (-0.34 to -0.2) | | (-0.01 to 0.24) | (-0.22 to 0.03) | (-0.34 to -0.1) |  |
| **Hepatitis** | -0.1 | 0.12 | -0.25 | -0.02 | 0.05 | -0.17 | | -0.34 | 0.36 | -0.17 | Infectious diseases |
|  | (-0.14 to -0.07) | (0.09 to 0.16) | (-0.28 to -0.22) | (-0.1 to 0.06) | (-0.03 to 0.13) | (-0.25 to -0.09) | | (-0.44 to -0.22) | (0.25 to 0.47) | (-0.29 to -0.04) |  |
| **HIV AIDS** | -0.31 | 0.38 | -0.21 | -0.1 | 0.16 | -0.19 | | -0.4 | 0.46 | -0.25 | Infectious diseases |
|  | (-0.34 to -0.27) | (0.35 to 0.41) | (-0.24 to -0.17) | (-0.18 to -0.03) | (0.08 to 0.23) | (-0.27 to -0.11) | | (-0.5 to -0.29) | (0.35 to 0.55) | (-0.37 to -0.13) |  |
| **Lower respiratory infections** | 0.13 | -0.05 | 0.04 | -0.01 | 0.07 | -0.1 | | 0.08 | -0.03 | -0.02 | Infectious diseases |
|  | (0.1 to 0.17) | (-0.08 to -0.01) | (0 to 0.07) | (-0.08 to 0.07) | (-0.01 to 0.15) | (-0.18 to -0.02) | | (-0.05 to 0.2) | (-0.16 to 0.1) | (-0.15 to 0.11) |  |
| **Meningitis** | -0.17 | 0.27 | -0.16 | -0.25 | 0.31 | -0.34 | | -0.27 | 0.34 | -0.07 | Infectious diseases |
|  | (-0.21 to -0.14) | (0.23 to 0.3) | (-0.19 to -0.13) | (-0.32 to -0.18) | (0.24 to 0.38) | (-0.41 to -0.26) | | (-0.39 to -0.15) | (0.23 to 0.45) | (-0.2 to 0.05) |  |
| **Tuberculosis** | -0.36 | 0.44 | -0.24 | -0.34 | 0.41 | -0.38 | | -0.45 | 0.5 | -0.19 | Infectious diseases |
|  | (-0.39 to -0.33) | (0.41 to 0.46) | (-0.27 to -0.21) | (-0.41 to -0.27) | (0.35 to 0.48) | (-0.44 to -0.31) | | (-0.54 to -0.34) | (0.4 to 0.59) | (-0.31 to -0.06) |  |
| **Years of Potential Life Lost Rate** | 0.17 | -0.09 | 0.21 | 0.05 | 0.01 | 0.07 | | 0.14 | -0.09 | 0.39 | Health Outcomes |
|  | (0.13 to 0.2) | (-0.13 to -0.06) | (0.18 to 0.25) | (-0.03 to 0.14) | (-0.07 to 0.1) | (-0.01 to 0.15) | | (0.02 to 0.27) | (-0.21 to 0.04) | (0.28 to 0.49) |  |
| **YPLL Rate (Black)** | 0.07 | -0.06 | 0.08 | -0.03 | 0.07 | 0.31 | | 0 | 0 | 0.32 | Health Outcomes |
|  | (0.02 to 0.12) | (-0.12 to -0.01) | (0.03 to 0.13) | (-0.16 to 0.09) | (-0.06 to 0.19) | (0.2 to 0.42) | | (-0.22 to 0.23) | (-0.23 to 0.23) | (0.1 to 0.51) |  |
| **YPLL Rate (White)** | 0.34 | -0.27 | 0.41 | 0.41 | -0.32 | 0.37 | | 0.31 | -0.25 | 0.66 | Health Outcomes |
|  | (0.29 to 0.38) | (-0.31 to -0.22) | (0.36 to 0.45) | (0.3 to 0.5) | (-0.43 to -0.21) | (0.26 to 0.47) | | (0.09 to 0.49) | (-0.44 to -0.03) | (0.52 to 0.77) |  |
| **% Fair/Poor** | -0.05 | 0.14 | 0.02 | -0.14 | 0.22 | -0.15 | | -0.03 | 0.06 | 0.35 | Health Outcomes |
|  | (-0.09 to -0.02) | (0.11 to 0.18) | (-0.02 to 0.05) | (-0.22 to -0.07) | (0.14 to 0.29) | (-0.22 to -0.07) | | (-0.15 to 0.1) | (-0.07 to 0.19) | (0.23 to 0.45) |  |
| **Physically Unhealthy Days** | 0.04 | 0.02 | 0.12 | -0.19 | 0.25 | -0.05 | | 0.07 | -0.05 | 0.37 | Health Outcomes |
|  | (0.01 to 0.08) | (-0.01 to 0.06) | (0.09 to 0.16) | (-0.26 to -0.11) | (0.18 to 0.33) | (-0.13 to 0.03) | | (-0.06 to 0.19) | (-0.17 to 0.08) | (0.25 to 0.47) |  |
| **Mentally Unhealthy Days** | 0.02 | 0.04 | 0.1 | -0.23 | 0.31 | -0.11 | | 0.12 | -0.08 | 0.25 | Health Outcomes |
|  | (-0.01 to 0.06) | (0 to 0.07) | (0.07 to 0.14) | (-0.31 to -0.16) | (0.24 to 0.38) | (-0.19 to -0.03) | | (-0.01 to 0.24) | (-0.21 to 0.05) | (0.13 to 0.37) |  |
| **% LBW** | -0.16 | 0.25 | -0.1 | -0.22 | 0.32 | -0.29 | | -0.16 | 0.21 | -0.05 | Health Outcomes |
|  | (-0.2 to -0.13) | (0.22 to 0.29) | (-0.14 to -0.07) | (-0.3 to -0.14) | (0.25 to 0.39) | (-0.36 to -0.21) | | (-0.28 to -0.03) | (0.08 to 0.33) | (-0.17 to 0.08) |  |
| **Life Expectancy** | -0.23 | 0.15 | -0.23 | -0.08 | 0.01 | -0.13 | | -0.19 | 0.15 | -0.45 | Health Outcomes |
|  | (-0.26 to -0.19) | (0.12 to 0.19) | (-0.26 to -0.19) | (-0.16 to 0) | (-0.07 to 0.09) | (-0.21 to -0.05) | | (-0.31 to -0.06) | (0.02 to 0.27) | (-0.55 to -0.34) |  |
| **Life Expectancy (Black)** | -0.15 | 0.09 | -0.2 | -0.02 | -0.03 | -0.15 | | 0 | -0.03 | -0.16 | Health Outcomes |
|  | (-0.2 to -0.1) | (0.04 to 0.14) | (-0.25 to -0.15) | (-0.14 to 0.1) | (-0.15 to 0.09) | (-0.27 to -0.03) | | (-0.21 to 0.21) | (-0.24 to 0.18) | (-0.36 to 0.05) |  |
| **Life Expectancy (White)** | -0.42 | 0.33 | -0.38 | -0.37 | 0.28 | -0.38 | | -0.29 | 0.23 | -0.71 | Health Outcomes |
|  | (-0.46 to -0.38) | (0.29 to 0.38) | (-0.42 to -0.34) | (-0.46 to -0.27) | (0.17 to 0.37) | (-0.47 to -0.28) | | (-0.45 to -0.11) | (0.05 to 0.4) | (-0.79 to -0.6) |  |
| **Age-Adjusted Mortality** | 0.2 | -0.12 | 0.21 | 0.09 | -0.02 | 0.08 | | 0.17 | -0.12 | 0.44 | Health Outcomes |
|  | (0.16 to 0.23) | (-0.15 to -0.08) | (0.17 to 0.24) | (0.01 to 0.16) | (-0.1 to 0.06) | (0 to 0.16) | | (0.04 to 0.29) | (-0.24 to 0.01) | (0.34 to 0.54) |  |
| **Age-Adjusted Mortality (Black)** | 0.06 | -0.06 | 0.04 | 0.09 | -0.07 | 0.38 | | 0.24 | -0.22 | 0.42 | Health Outcomes |
|  | (0 to 0.11) | (-0.11 to 0) | (-0.01 to 0.09) | (-0.03 to 0.22) | (-0.19 to 0.06) | (0.27 to 0.48) | | (0.01 to 0.44) | (-0.43 to 0) | (0.21 to 0.59) |  |
| **Age-Adjusted Mortality (White)** | 0.36 | -0.28 | 0.42 | 0.41 | -0.34 | 0.4 | | 0.26 | -0.21 | 0.72 | Health Outcomes |
|  | (0.32 to 0.4) | (-0.33 to -0.24) | (0.38 to 0.46) | (0.31 to 0.51) | (-0.45 to -0.23) | (0.3 to 0.5) | | (0.05 to 0.46) | (-0.41 to 0.01) | (0.6 to 0.81) |  |
| **Child Mortality Rate** | 0.13 | -0.07 | 0.16 | 0.02 | 0.03 | 0.06 | | 0.06 | -0.04 | 0.22 | Health Outcomes |
|  | (0.09 to 0.18) | (-0.12 to -0.03) | (0.11 to 0.2) | (-0.08 to 0.11) | (-0.07 to 0.13) | (-0.04 to 0.16) | | (-0.09 to 0.2) | (-0.18 to 0.11) | (0.07 to 0.35) |  |
| **Infant Mortality Rate** | 0.16 | -0.09 | 0.28 | 0.01 | 0.06 | 0.15 | | -0.04 | 0.07 | 0.31 | Health Outcomes |
|  | (0.11 to 0.21) | (-0.14 to -0.03) | (0.23 to 0.33) | (-0.11 to 0.13) | (-0.06 to 0.18) | (0.04 to 0.26) | | (-0.22 to 0.15) | (-0.11 to 0.25) | (0.14 to 0.47) |  |
| **% Frequent Physical Distress** | 0 | 0.07 | 0.11 | -0.19 | 0.26 | -0.08 | | 0.02 | 0 | 0.37 | Health Outcomes |
|  | (-0.04 to 0.03) | (0.04 to 0.11) | (0.07 to 0.14) | (-0.27 to -0.12) | (0.19 to 0.33) | (-0.16 to 0) | | (-0.11 to 0.14) | (-0.12 to 0.13) | (0.25 to 0.47) |  |
| **% Frequent Mental Distress** | 0 | 0.07 | 0.12 | -0.22 | 0.3 | -0.1 | | 0.04 | -0.01 | 0.34 | Health Outcomes |
|  | (-0.04 to 0.03) | (0.04 to 0.11) | (0.09 to 0.16) | (-0.29 to -0.14) | (0.23 to 0.37) | (-0.17 to -0.02) | | (-0.09 to 0.17) | (-0.14 to 0.12) | (0.22 to 0.45) |  |
| **HIV Prevalence Rate** | -0.4 | 0.47 | -0.25 | -0.2 | 0.29 | -0.31 | | -0.44 | 0.49 | -0.27 | Health Outcomes |
|  | (-0.44 to -0.37) | (0.44 to 0.5) | (-0.28 to -0.21) | (-0.29 to -0.11) | (0.21 to 0.38) | (-0.39 to -0.23) | | (-0.54 to -0.32) | (0.38 to 0.59) | (-0.39 to -0.13) |  |
| **diabetes_crude** | 0.17 | -0.1 | 0.23 | 0.13 | -0.07 | 0.17 | | 0.39 | -0.37 | 0.38 | Health Behaviors |
|  | (0.14 to 0.2) | (-0.13 to -0.06) | (0.2 to 0.26) | (0.05 to 0.21) | (-0.15 to 0.01) | (0.09 to 0.25) | | (0.28 to 0.49) | (-0.47 to -0.25) | (0.26 to 0.48) |  |
| **obesity_crude** | 0.16 | -0.1 | 0.28 | 0.2 | -0.17 | 0.33 | | 0.26 | -0.26 | 0.38 | Health Behaviors |
|  | (0.12 to 0.19) | (-0.14 to -0.07) | (0.24 to 0.31) | (0.12 to 0.27) | (-0.25 to -0.09) | (0.25 to 0.39) | | (0.14 to 0.38) | (-0.38 to -0.14) | (0.27 to 0.49) |  |
| **physical_inactivity_crude** | 0.36 | -0.28 | 0.3 | 0.38 | -0.31 | 0.2 | | 0.46 | -0.43 | 0.53 | Health Behaviors |
|  | (0.33 to 0.39) | (-0.31 to -0.25) | (0.26 to 0.33) | (0.31 to 0.45) | (-0.38 to -0.24) | (0.13 to 0.28) | | (0.35 to 0.55) | (-0.53 to -0.32) | (0.44 to 0.62) |  |
| **% Smokers** | 0.12 | -0.04 | 0.35 | -0.03 | 0.09 | 0.14 | | 0.04 | -0.04 | 0.43 | Health Behaviors |
|  | (0.09 to 0.16) | (-0.07 to 0) | (0.32 to 0.38) | (-0.11 to 0.05) | (0.01 to 0.17) | (0.06 to 0.22) | | (-0.09 to 0.17) | (-0.16 to 0.09) | (0.32 to 0.53) |  |
| **Food Environment Index** | 0.06 | -0.1 | 0.06 | -0.01 | -0.02 | 0.16 | | 0.17 | -0.2 | -0.19 | Health Behaviors |
|  | (0.02 to 0.09) | (-0.13 to -0.06) | (0.03 to 0.1) | (-0.09 to 0.07) | (-0.1 to 0.06) | (0.08 to 0.24) | | (0.05 to 0.3) | (-0.32 to -0.07) | (-0.31 to -0.06) |  |
| **% Excessive Drinking** | -0.16 | 0.11 | 0.07 | -0.12 | 0.05 | 0.18 | | -0.34 | 0.3 | -0.25 | Health Behaviors |
|  | (-0.19 to -0.12) | (0.07 to 0.14) | (0.03 to 0.1) | (-0.2 to -0.04) | (-0.03 to 0.13) | (0.1 to 0.26) | | (-0.44 to -0.22) | (0.18 to 0.41) | (-0.36 to -0.12) |  |
| **Teen Birth Rate** | 0.22 | -0.15 | 0.1 | 0.11 | -0.07 | 0 | | 0.19 | -0.16 | 0.42 | Health Behaviors |
|  | (0.19 to 0.26) | (-0.18 to -0.11) | (0.07 to 0.14) | (0.03 to 0.19) | (-0.15 to 0.01) | (-0.08 to 0.08) | | (0.07 to 0.31) | (-0.28 to -0.03) | (0.31 to 0.52) |  |
| **% Food Insecure** | -0.14 | 0.21 | -0.07 | -0.21 | 0.28 | -0.23 | | -0.15 | 0.17 | 0.2 | Health Behaviors |
|  | (-0.17 to -0.1) | (0.18 to 0.24) | (-0.11 to -0.04) | (-0.28 to -0.13) | (0.21 to 0.35) | (-0.3 to -0.15) | | (-0.27 to -0.02) | (0.05 to 0.29) | (0.08 to 0.32) |  |
| **Drug Overdose Mortality Rate** | 0.15 | -0.13 | 0.3 | 0.12 | -0.07 | 0.11 | | 0.02 | 0.06 | 0.05 | Health Behaviors |
|  | (0.11 to 0.2) | (-0.18 to -0.09) | (0.25 to 0.34) | (0.02 to 0.21) | (-0.17 to 0.03) | (0.01 to 0.21) | | (-0.13 to 0.16) | (-0.09 to 0.2) | (-0.1 to 0.19) |  |
| **MV Mortality Rate** | 0.37 | -0.32 | 0.21 | 0.31 | -0.28 | 0.21 | | 0.36 | -0.34 | 0.54 | Health Behaviors |
|  | (0.33 to 0.4) | (-0.35 to -0.28) | (0.17 to 0.24) | (0.23 to 0.39) | (-0.36 to -0.2) | (0.13 to 0.29) | | (0.25 to 0.47) | (-0.45 to -0.22) | (0.44 to 0.62) |  |
| **% Insufficient Sleep** | -0.16 | 0.25 | 0.03 | -0.27 | 0.36 | -0.03 | | 0.09 | -0.05 | 0.14 | Health Behaviors |
|  | (-0.19 to -0.12) | (0.22 to 0.28) | (-0.01 to 0.06) | (-0.35 to -0.2) | (0.29 to 0.43) | (-0.11 to 0.05) | | (-0.04 to 0.22) | (-0.17 to 0.08) | (0.02 to 0.26) |  |
| **opioid_prescribing_rate** | 0.13 | -0.1 | -0.02 | 0.1 | -0.04 | -0.06 | | 0.13 | -0.13 | 0.16 | Health Behaviors |
|  | (0.09 to 0.16) | (-0.13 to -0.06) | (-0.05 to 0.02) | (0.02 to 0.18) | (-0.12 to 0.04) | (-0.14 to 0.02) | | (0 to 0.25) | (-0.25 to 0) | (0.03 to 0.28) |  |
| **% 65 and over** | 0.28 | -0.27 | 0.34 | 0.35 | -0.32 | 0.26 | | 0.38 | -0.37 | 0.4 | Demographic |
|  | (0.25 to 0.32) | (-0.3 to -0.24) | (0.3 to 0.37) | (0.28 to 0.42) | (-0.39 to -0.24) | (0.18 to 0.33) | | (0.26 to 0.48) | (-0.47 to -0.25) | (0.29 to 0.5) |  |
| **% Non-Hispanic White** | 0.53 | -0.58 | 0.44 | 0.51 | -0.55 | 0.48 | | 0.59 | -0.64 | 0.35 | Demographic |
|  | (0.5 to 0.56) | (-0.61 to -0.56) | (0.41 to 0.47) | (0.45 to 0.57) | (-0.61 to -0.5) | (0.42 to 0.54) | | (0.5 to 0.66) | (-0.71 to -0.56) | (0.23 to 0.45) |  |
| **% Rural** | 0.49 | -0.47 | 0.44 | 0.47 | -0.47 | 0.49 | | 0.45 | -0.46 | 0.59 | Demographic |
|  | (0.46 to 0.52) | (-0.49 to -0.44) | (0.41 to 0.47) | (0.41 to 0.53) | (-0.53 to -0.4) | (0.42 to 0.55) | | (0.34 to 0.54) | (-0.56 to -0.36) | (0.5 to 0.66) |  |
| **Alcohol use disorders** | -0.23 | 0.18 | -0.02 | -0.3 | 0.27 | -0.02 | | -0.4 | 0.4 | 0.09 | Deaths of Despair |
|  | (-0.26 to -0.19) | (0.15 to 0.21) | (-0.06 to 0.01) | (-0.37 to -0.22) | (0.2 to 0.34) | (-0.1 to 0.06) | | (-0.5 to -0.28) | (0.29 to 0.5) | (-0.04 to 0.21) |  |
| **Drug use disorders** | 0.11 | -0.08 | 0.09 | -0.15 | 0.2 | -0.1 | | -0.06 | 0.11 | 0.04 | Deaths of Despair |
|  | (0.07 to 0.14) | (-0.12 to -0.05) | (0.06 to 0.12) | (-0.23 to -0.07) | (0.12 to 0.27) | (-0.18 to -0.02) | | (-0.18 to 0.07) | (-0.02 to 0.24) | (-0.09 to 0.17) |  |
| **Interpersonal violence** | -0.29 | 0.37 | -0.14 | -0.3 | 0.37 | -0.3 | | -0.36 | 0.42 | -0.09 | Deaths of Despair |
|  | (-0.32 to -0.26) | (0.34 to 0.4) | (-0.17 to -0.1) | (-0.37 to -0.22) | (0.3 to 0.43) | (-0.37 to -0.22) | | (-0.47 to -0.24) | (0.31 to 0.52) | (-0.21 to 0.04) |  |
| **Self-harm** | 0.22 | -0.26 | 0.13 | 0.12 | -0.12 | 0.16 | | 0.18 | -0.15 | 0.56 | Deaths of Despair |
|  | (0.19 to 0.25) | (-0.3 to -0.23) | (0.09 to 0.16) | (0.04 to 0.19) | (-0.2 to -0.04) | (0.09 to 0.24) | | (0.05 to 0.3) | (-0.27 to -0.03) | (0.47 to 0.64) |  |
| **% With Access** | -0.37 | 0.32 | -0.25 | -0.41 | 0.39 | -0.21 | | -0.28 | 0.28 | -0.38 | Clinical Care |
|  | (-0.4 to -0.34) | (0.28 to 0.35) | (-0.28 to -0.22) | (-0.48 to -0.35) | (0.32 to 0.46) | (-0.28 to -0.13) | | (-0.4 to -0.16) | (0.16 to 0.4) | (-0.48 to -0.26) |  |
| **PCP Rate** | -0.35 | 0.31 | -0.27 | -0.3 | 0.27 | -0.21 | | -0.35 | 0.34 | -0.38 | Clinical Care |
|  | (-0.38 to -0.32) | (0.28 to 0.35) | (-0.3 to -0.24) | (-0.37 to -0.23) | (0.2 to 0.35) | (-0.28 to -0.13) | | (-0.46 to -0.23) | (0.22 to 0.45) | (-0.48 to -0.26) |  |
| **Dentist Rate** | -0.38 | 0.34 | -0.23 | -0.35 | 0.32 | -0.24 | | -0.47 | 0.49 | -0.41 | Clinical Care |
|  | (-0.41 to -0.35) | (0.31 to 0.37) | (-0.27 to -0.2) | (-0.42 to -0.28) | (0.25 to 0.39) | (-0.31 to -0.16) | | (-0.57 to -0.37) | (0.39 to 0.58) | (-0.51 to -0.29) |  |
| **MHP Rate** | -0.4 | 0.35 | -0.23 | -0.47 | 0.46 | -0.26 | | -0.52 | 0.5 | -0.35 | Clinical Care |
|  | (-0.43 to -0.37) | (0.32 to 0.39) | (-0.26 to -0.19) | (-0.53 to -0.41) | (0.39 to 0.52) | (-0.33 to -0.18) | | (-0.61 to -0.42) | (0.4 to 0.59) | (-0.46 to -0.23) |  |
| **Preventable Hosp. Rate** | 0.13 | -0.05 | 0.14 | -0.05 | 0.08 | 0.16 | | -0.02 | 0.06 | 0.15 | Clinical Care |
|  | (0.09 to 0.16) | (-0.09 to -0.02) | (0.11 to 0.17) | (-0.12 to 0.03) | (0 to 0.16) | (0.08 to 0.23) | | (-0.15 to 0.1) | (-0.07 to 0.18) | (0.03 to 0.27) |  |
| **% Screened** | -0.17 | 0.16 | 0.09 | -0.11 | 0.1 | 0.12 | | -0.01 | -0.03 | -0.1 | Clinical Care |
|  | (-0.21 to -0.14) | (0.12 to 0.19) | (0.06 to 0.13) | (-0.19 to -0.03) | (0.02 to 0.18) | (0.04 to 0.2) | | (-0.14 to 0.12) | (-0.15 to 0.1) | (-0.23 to 0.03) |  |
| **% Vaccinated** | -0.23 | 0.21 | -0.1 | -0.35 | 0.35 | -0.26 | | -0.34 | 0.34 | -0.51 | Clinical Care |
|  | (-0.26 to -0.19) | (0.18 to 0.25) | (-0.13 to -0.06) | (-0.42 to -0.28) | (0.28 to 0.42) | (-0.33 to -0.18) | | (-0.45 to -0.22) | (0.23 to 0.45) | (-0.6 to -0.41) |  |
| **Aortic aneurysm** | 0.29 | -0.29 | 0.45 | 0.15 | -0.17 | 0.51 | | 0.2 | -0.22 | 0.45 | Cardiovascular diseases |
|  | (0.25 to 0.32) | (-0.32 to -0.26) | (0.42 to 0.48) | (0.07 to 0.23) | (-0.25 to -0.09) | (0.45 to 0.57) | | (0.07 to 0.31) | (-0.34 to -0.1) | (0.34 to 0.55) |  |
| **Atrial fibrillation & flutter** | 0.06 | -0.12 | -0.01 | 0.04 | -0.06 | 0.15 | | -0.06 | 0.04 | 0.04 | Cardiovascular diseases |
|  | (0.03 to 0.1) | (-0.16 to -0.09) | (-0.05 to 0.02) | (-0.04 to 0.12) | (-0.14 to 0.02) | (0.07 to 0.23) | | (-0.19 to 0.07) | (-0.09 to 0.17) | (-0.09 to 0.16) |  |
| **Cardiomyopathy & myocarditis** | -0.2 | 0.27 | -0.05 | -0.25 | 0.34 | -0.17 | | 0 | 0.04 | -0.09 | Cardiovascular diseases |
|  | (-0.23 to -0.16) | (0.24 to 0.31) | (-0.08 to -0.01) | (-0.32 to -0.17) | (0.27 to 0.41) | (-0.24 to -0.09) | | (-0.13 to 0.13) | (-0.09 to 0.16) | (-0.21 to 0.04) |  |
| **Cardiovascular diseases** | 0.23 | -0.14 | 0.2 | 0.06 | 0.01 | 0.18 | | 0.26 | -0.22 | 0.42 | Cardiovascular diseases |
|  | (0.2 to 0.26) | (-0.17 to -0.11) | (0.17 to 0.24) | (-0.01 to 0.14) | (-0.07 to 0.09) | (0.11 to 0.26) | | (0.14 to 0.38) | (-0.34 to -0.09) | (0.3 to 0.52) |  |
| **Cerebrovascular disease** | 0.12 | -0.06 | 0.05 | -0.07 | 0.11 | 0.01 | | -0.01 | 0.02 | 0.06 | Cardiovascular diseases |
|  | (0.09 to 0.16) | (-0.1 to -0.03) | (0.01 to 0.08) | (-0.15 to 0.01) | (0.03 to 0.19) | (-0.07 to 0.09) | | (-0.14 to 0.12) | (-0.1 to 0.15) | (-0.06 to 0.19) |  |
| **Endocarditis** | 0.05 | -0.06 | 0.04 | 0.14 | -0.11 | 0.04 | | 0.08 | -0.05 | -0.03 | Cardiovascular diseases |
|  | (0.02 to 0.09) | (-0.09 to -0.02) | (0 to 0.07) | (0.06 to 0.22) | (-0.19 to -0.03) | (-0.04 to 0.12) | | (-0.05 to 0.21) | (-0.18 to 0.08) | (-0.16 to 0.1) |  |
| **Hemorrhagic stroke** | 0 | 0.09 | 0.01 | -0.14 | 0.21 | -0.05 | | 0.02 | 0.03 | 0.12 | Cardiovascular diseases |
|  | (-0.03 to 0.04) | (0.06 to 0.13) | (-0.03 to 0.04) | (-0.22 to -0.06) | (0.13 to 0.29) | (-0.13 to 0.03) | | (-0.11 to 0.14) | (-0.1 to 0.15) | (0 to 0.25) |  |
| **Hypertensive heart disease** | -0.13 | 0.18 | -0.13 | -0.19 | 0.23 | -0.22 | | -0.31 | 0.32 | -0.12 | Cardiovascular diseases |
|  | (-0.16 to -0.09) | (0.15 to 0.22) | (-0.17 to -0.1) | (-0.26 to -0.11) | (0.15 to 0.3) | (-0.3 to -0.14) | | (-0.42 to -0.19) | (0.2 to 0.43) | (-0.24 to 0.01) |  |
| **Ischemic heart disease** | 0.28 | -0.2 | 0.26 | 0.14 | -0.06 | 0.24 | | 0.33 | -0.29 | 0.47 | Cardiovascular diseases |
|  | (0.25 to 0.31) | (-0.23 to -0.16) | (0.22 to 0.29) | (0.06 to 0.21) | (-0.14 to 0.01) | (0.17 to 0.32) | | (0.21 to 0.44) | (-0.4 to -0.17) | (0.36 to 0.56) |  |
| **Ischemic stroke** | 0.17 | -0.13 | 0.06 | -0.03 | 0.07 | 0.03 | | -0.02 | 0.02 | 0.04 | Cardiovascular diseases |
|  | (0.13 to 0.2) | (-0.17 to -0.1) | (0.03 to 0.1) | (-0.11 to 0.05) | (-0.01 to 0.14) | (-0.05 to 0.11) | | (-0.15 to 0.11) | (-0.11 to 0.15) | (-0.09 to 0.16) |  |
| **Other cardiovascular** | 0.01 | 0 | 0.1 | 0.1 | -0.09 | 0.17 | | 0.08 | -0.06 | -0.01 | Cardiovascular diseases |
|  | (-0.02 to 0.05) | (-0.03 to 0.04) | (0.07 to 0.14) | (0.03 to 0.18) | (-0.16 to -0.01) | (0.09 to 0.24) | | (-0.05 to 0.2) | (-0.19 to 0.06) | (-0.13 to 0.12) |  |
| **Peripheral vascular disease** | 0.05 | 0.01 | 0.13 | -0.08 | 0.1 | 0.1 | | 0.07 | -0.06 | 0.12 | Cardiovascular diseases |
|  | (0.01 to 0.08) | (-0.02 to 0.05) | (0.09 to 0.16) | (-0.15 to 0) | (0.02 to 0.18) | (0.02 to 0.17) | | (-0.06 to 0.19) | (-0.19 to 0.07) | (-0.01 to 0.24) |  |
| **Rheumatic heart disease** | 0.13 | -0.1 | -0.03 | 0.29 | -0.31 | 0.29 | | 0.12 | -0.12 | 0.29 | Cardiovascular diseases |
|  | (0.1 to 0.17) | (-0.14 to -0.07) | (-0.06 to 0.01) | (0.21 to 0.36) | (-0.38 to -0.24) | (0.21 to 0.36) | | (-0.01 to 0.24) | (-0.24 to 0.01) | (0.17 to 0.4) |  |
| **Acute lymphoid leukemia** | 0.14 | -0.07 | -0.02 | 0.05 | -0.04 | 0.05 | | 0.26 | -0.21 | 0.46 | Cancers |
|  | (0.1 to 0.17) | (-0.11 to -0.04) | (-0.06 to 0.01) | (-0.03 to 0.13) | (-0.12 to 0.04) | (-0.03 to 0.13) | | (0.13 to 0.37) | (-0.33 to -0.09) | (0.36 to 0.56) |  |
| **Acute myeloid leukemia** | 0.26 | -0.24 | 0.41 | 0.12 | -0.13 | 0.48 | | 0.15 | -0.16 | 0.41 | Cancers |
|  | (0.23 to 0.3) | (-0.27 to -0.21) | (0.38 to 0.44) | (0.04 to 0.2) | (-0.21 to -0.05) | (0.42 to 0.54) | | (0.03 to 0.28) | (-0.28 to -0.03) | (0.3 to 0.51) |  |
| **Bladder cancer** | 0.11 | -0.12 | 0.34 | -0.01 | 0.03 | 0.24 | | 0.19 | -0.22 | 0.29 | Cancers |
|  | (0.08 to 0.15) | (-0.15 to -0.08) | (0.3 to 0.37) | (-0.09 to 0.07) | (-0.05 to 0.11) | (0.17 to 0.32) | | (0.07 to 0.31) | (-0.33 to -0.09) | (0.17 to 0.4) |  |
| **Brain & nervous system cancer** | 0.48 | -0.48 | 0.29 | 0.31 | -0.35 | 0.35 | | 0.08 | -0.15 | 0.35 | Cancers |
|  | (0.45 to 0.51) | (-0.5 to -0.45) | (0.26 to 0.32) | (0.24 to 0.38) | (-0.42 to -0.28) | (0.28 to 0.42) | | (-0.05 to 0.2) | (-0.27 to -0.02) | (0.24 to 0.46) |  |
| **Breast cancer** | -0.04 | 0.12 | -0.03 | -0.11 | 0.18 | -0.12 | | 0 | 0.05 | 0.1 | Cancers |
|  | (-0.07 to 0) | (0.08 to 0.15) | (-0.07 to 0) | (-0.19 to -0.03) | (0.1 to 0.25) | (-0.2 to -0.04) | | (-0.12 to 0.13) | (-0.07 to 0.18) | (-0.03 to 0.22) |  |
| **Cervical cancer** | -0.04 | 0.14 | 0.06 | -0.13 | 0.22 | -0.09 | | 0.1 | -0.03 | 0.26 | Cancers |
|  | (-0.07 to 0) | (0.11 to 0.17) | (0.02 to 0.09) | (-0.21 to -0.05) | (0.14 to 0.29) | (-0.17 to -0.01) | | (-0.03 to 0.22) | (-0.16 to 0.1) | (0.14 to 0.38) |  |
| **Chronic lymphoid leukemia** | 0.38 | -0.38 | 0.5 | 0.24 | -0.29 | 0.54 | | 0.36 | -0.37 | 0.33 | Cancers |
|  | (0.35 to 0.41) | (-0.41 to -0.35) | (0.47 to 0.52) | (0.17 to 0.32) | (-0.36 to -0.22) | (0.48 to 0.59) | | (0.24 to 0.46) | (-0.47 to -0.25) | (0.22 to 0.44) |  |
| **Chronic myeloid leukemia** | 0.22 | -0.16 | 0.17 | -0.04 | 0.05 | 0.22 | | 0.07 | -0.08 | 0.31 | Cancers |
|  | (0.19 to 0.25) | (-0.2 to -0.13) | (0.14 to 0.2) | (-0.12 to 0.04) | (-0.03 to 0.13) | (0.15 to 0.3) | | (-0.05 to 0.2) | (-0.21 to 0.05) | (0.19 to 0.42) |  |
| **Colon & rectum cancer** | 0.19 | -0.11 | 0.32 | 0.22 | -0.18 | 0.23 | | 0.24 | -0.19 | 0.39 | Cancers |
|  | (0.16 to 0.23) | (-0.14 to -0.07) | (0.29 to 0.35) | (0.14 to 0.29) | (-0.26 to -0.11) | (0.15 to 0.3) | | (0.11 to 0.35) | (-0.31 to -0.06) | (0.27 to 0.49) |  |
| **Esophageal cancer** | -0.03 | 0.04 | 0.41 | 0.01 | -0.02 | 0.41 | | 0.21 | -0.22 | 0.47 | Cancers |
|  | (-0.06 to 0.01) | (0.01 to 0.08) | (0.38 to 0.44) | (-0.07 to 0.08) | (-0.1 to 0.06) | (0.34 to 0.47) | | (0.09 to 0.33) | (-0.33 to -0.09) | (0.37 to 0.57) |  |
| **Gallbladder & biliary tract** | -0.15 | 0.15 | 0.25 | -0.18 | 0.13 | 0.27 | | -0.11 | 0.15 | 0.17 | Cancers |
|  | (-0.18 to -0.11) | (0.11 to 0.18) | (0.22 to 0.29) | (-0.25 to -0.1) | (0.05 to 0.21) | (0.19 to 0.34) | | (-0.23 to 0.02) | (0.02 to 0.27) | (0.04 to 0.29) |  |
| **Hodgkin lymphoma** | 0.06 | 0.03 | 0.17 | 0.12 | -0.11 | 0.33 | | 0.29 | -0.28 | 0.39 | Cancers |
|  | (0.02 to 0.09) | (-0.01 to 0.06) | (0.14 to 0.2) | (0.04 to 0.19) | (-0.18 to -0.03) | (0.26 to 0.4) | | (0.17 to 0.4) | (-0.39 to -0.16) | (0.28 to 0.49) |  |
| **Kidney cancer** | 0.35 | -0.3 | 0.35 | 0.11 | -0.14 | 0.37 | | 0.07 | -0.07 | 0.46 | Cancers |
|  | (0.32 to 0.38) | (-0.33 to -0.27) | (0.32 to 0.38) | (0.03 to 0.19) | (-0.21 to -0.06) | (0.3 to 0.43) | | (-0.05 to 0.2) | (-0.19 to 0.06) | (0.35 to 0.55) |  |
| **Larynx cancer** | 0 | 0.12 | 0.18 | -0.11 | 0.21 | -0.07 | | 0 | 0.03 | 0.34 | Cancers |
|  | (-0.04 to 0.03) | (0.08 to 0.15) | (0.15 to 0.22) | (-0.19 to -0.03) | (0.13 to 0.28) | (-0.15 to 0.01) | | (-0.13 to 0.12) | (-0.1 to 0.16) | (0.22 to 0.44) |  |
| **Leukemia** | 0.34 | -0.31 | 0.44 | 0.17 | -0.19 | 0.51 | | 0.26 | -0.26 | 0.44 | Cancers |
|  | (0.31 to 0.37) | (-0.34 to -0.28) | (0.41 to 0.47) | (0.09 to 0.25) | (-0.27 to -0.12) | (0.45 to 0.57) | | (0.14 to 0.37) | (-0.38 to -0.14) | (0.33 to 0.54) |  |
| **Lip & oral cavity cancer** | 0.04 | 0.05 | 0.01 | -0.03 | 0.1 | -0.04 | | -0.02 | 0 | 0.44 | Cancers |
|  | (0.01 to 0.08) | (0.02 to 0.09) | (-0.03 to 0.04) | (-0.11 to 0.05) | (0.02 to 0.17) | (-0.12 to 0.04) | | (-0.14 to 0.11) | (-0.13 to 0.12) | (0.33 to 0.54) |  |
| **Liver cancer** | -0.18 | 0.25 | -0.13 | -0.28 | 0.32 | -0.12 | | -0.26 | 0.32 | 0.24 | Cancers |
|  | (-0.21 to -0.14) | (0.22 to 0.28) | (-0.16 to -0.1) | (-0.35 to -0.2) | (0.25 to 0.39) | (-0.2 to -0.04) | | (-0.38 to -0.14) | (0.2 to 0.43) | (0.12 to 0.36) |  |
| **Malignant skin melanoma** | 0.54 | -0.56 | 0.15 | 0.46 | -0.44 | -0.06 | | 0.34 | -0.36 | 0.14 | Cancers |
|  | (0.51 to 0.56) | (-0.59 to -0.54) | (0.11 to 0.18) | (0.4 to 0.52) | (-0.5 to -0.37) | (-0.13 to 0.02) | | (0.22 to 0.45) | (-0.46 to -0.24) | (0.02 to 0.27) |  |
| **Mesothelioma** | -0.05 | 0.01 | 0.13 | -0.16 | 0.13 | 0.27 | | -0.08 | 0.06 | 0.01 | Cancers |
|  | (-0.09 to -0.02) | (-0.02 to 0.05) | (0.1 to 0.17) | (-0.24 to -0.08) | (0.05 to 0.21) | (0.19 to 0.34) | | (-0.21 to 0.04) | (-0.07 to 0.19) | (-0.12 to 0.14) |  |
| **Multiple myeloma** | -0.21 | 0.27 | 0 | -0.27 | 0.3 | 0.03 | | -0.2 | 0.21 | 0.1 | Cancers |
|  | (-0.24 to -0.17) | (0.23 to 0.3) | (-0.04 to 0.03) | (-0.34 to -0.2) | (0.22 to 0.37) | (-0.05 to 0.11) | | (-0.32 to -0.08) | (0.08 to 0.33) | (-0.03 to 0.22) |  |
| **Nasopharynx cancer** | -0.24 | 0.34 | -0.11 | -0.25 | 0.34 | -0.22 | | -0.31 | 0.35 | 0.16 | Cancers |
|  | (-0.28 to -0.21) | (0.3 to 0.37) | (-0.14 to -0.07) | (-0.32 to -0.18) | (0.27 to 0.41) | (-0.29 to -0.14) | | (-0.42 to -0.19) | (0.23 to 0.45) | (0.03 to 0.28) |  |
| **Neoplasms** | 0.18 | -0.09 | 0.29 | -0.01 | 0.06 | 0.2 | | 0.09 | -0.06 | 0.49 | Cancers |
|  | (0.14 to 0.21) | (-0.12 to -0.05) | (0.26 to 0.32) | (-0.09 to 0.07) | (-0.02 to 0.14) | (0.12 to 0.27) | | (-0.04 to 0.21) | (-0.19 to 0.07) | (0.38 to 0.58) |  |
| **Non-Hodgkin lymphoma** | 0.29 | -0.28 | 0.43 | 0.12 | -0.14 | 0.4 | | 0.38 | -0.37 | 0.23 | Cancers |
|  | (0.26 to 0.32) | (-0.32 to -0.25) | (0.4 to 0.45) | (0.04 to 0.2) | (-0.22 to -0.06) | (0.33 to 0.46) | | (0.26 to 0.48) | (-0.48 to -0.26) | (0.11 to 0.35) |  |
| **Non-melanoma skin cancer** | 0.34 | -0.31 | 0.02 | 0.13 | -0.08 | -0.21 | | 0.17 | -0.19 | 0.29 | Cancers |
|  | (0.3 to 0.37) | (-0.34 to -0.28) | (-0.01 to 0.06) | (0.05 to 0.21) | (-0.16 to -0.01) | (-0.28 to -0.13) | | (0.04 to 0.29) | (-0.31 to -0.06) | (0.17 to 0.41) |  |
| **Other neoplasms** | 0.03 | 0.03 | 0.28 | -0.13 | 0.12 | 0.39 | | -0.04 | 0.03 | 0.36 | Cancers |
|  | (-0.01 to 0.06) | (-0.01 to 0.06) | (0.25 to 0.31) | (-0.21 to -0.05) | (0.04 to 0.2) | (0.32 to 0.45) | | (-0.17 to 0.09) | (-0.1 to 0.16) | (0.24 to 0.46) |  |
| **Other pharynx cancer** | -0.15 | 0.25 | 0.05 | -0.21 | 0.29 | -0.05 | | -0.14 | 0.13 | 0.32 | Cancers |
|  | (-0.18 to -0.11) | (0.21 to 0.28) | (0.02 to 0.09) | (-0.28 to -0.13) | (0.21 to 0.36) | (-0.13 to 0.03) | | (-0.26 to -0.01) | (0 to 0.25) | (0.2 to 0.43) |  |
| **Ovarian cancer** | 0.02 | -0.03 | 0.07 | 0.06 | -0.06 | 0.21 | | 0.2 | -0.16 | 0.14 | Cancers |
|  | (-0.01 to 0.06) | (-0.06 to 0.01) | (0.03 to 0.1) | (-0.02 to 0.13) | (-0.13 to 0.02) | (0.14 to 0.29) | | (0.07 to 0.32) | (-0.28 to -0.03) | (0.01 to 0.26) |  |
| **Pancreatic cancer** | -0.12 | 0.19 | 0.11 | -0.25 | 0.29 | 0.16 | | -0.1 | 0.13 | 0.16 | Cancers |
|  | (-0.15 to -0.08) | (0.16 to 0.23) | (0.08 to 0.15) | (-0.32 to -0.17) | (0.21 to 0.36) | (0.08 to 0.23) | | (-0.23 to 0.03) | (0.01 to 0.26) | (0.04 to 0.28) |  |
| **Prostate cancer** | -0.22 | 0.26 | -0.05 | -0.23 | 0.22 | 0.13 | | -0.2 | 0.2 | 0.07 | Cancers |
|  | (-0.25 to -0.18) | (0.22 to 0.29) | (-0.08 to -0.01) | (-0.3 to -0.15) | (0.15 to 0.3) | (0.05 to 0.21) | | (-0.32 to -0.07) | (0.08 to 0.32) | (-0.06 to 0.2) |  |
| **Stomach cancer** | -0.35 | 0.44 | -0.16 | -0.47 | 0.52 | -0.13 | | -0.3 | 0.37 | 0.03 | Cancers |
|  | (-0.38 to -0.32) | (0.41 to 0.47) | (-0.2 to -0.13) | (-0.53 to -0.4) | (0.46 to 0.57) | (-0.21 to -0.05) | | (-0.41 to -0.18) | (0.25 to 0.47) | (-0.09 to 0.16) |  |
| **Testicular cancer** | 0.31 | -0.29 | 0.27 | 0.25 | -0.23 | 0.21 | | 0.42 | -0.41 | 0.52 | Cancers |
|  | (0.28 to 0.34) | (-0.32 to -0.25) | (0.24 to 0.3) | (0.17 to 0.32) | (-0.31 to -0.16) | (0.13 to 0.29) | | (0.31 to 0.52) | (-0.51 to -0.3) | (0.43 to 0.61) |  |
| **Thyroid cancer** | -0.1 | 0.1 | 0 | -0.17 | 0.13 | 0.19 | | -0.06 | 0.12 | 0.14 | Cancers |
|  | (-0.14 to -0.07) | (0.06 to 0.13) | (-0.03 to 0.04) | (-0.25 to -0.1) | (0.05 to 0.2) | (0.11 to 0.26) | | (-0.19 to 0.07) | (-0.01 to 0.24) | (0.01 to 0.26) |  |
| **Tracheal, bronchus, & lung** | 0.27 | -0.18 | 0.32 | 0.05 | 0.02 | 0.15 | | 0.1 | -0.09 | 0.51 | Cancers |
|  | (0.24 to 0.31) | (-0.22 to -0.15) | (0.29 to 0.36) | (-0.03 to 0.13) | (-0.06 to 0.1) | (0.08 to 0.23) | | (-0.03 to 0.23) | (-0.21 to 0.04) | (0.41 to 0.6) |  |
| **Uterine cancer** | -0.34 | 0.35 | 0.21 | -0.24 | 0.27 | 0.12 | | -0.18 | 0.22 | -0.04 | Cancers |
|  | (-0.37 to -0.31) | (0.32 to 0.38) | (0.18 to 0.25) | (-0.32 to -0.17) | (0.19 to 0.34) | (0.04 to 0.2) | | (-0.3 to -0.05) | (0.1 to 0.34) | (-0.17 to 0.09) |  |
