# Supplementary material for: Viewing the US presidential electoral map through the lens of public health
Source: PLoS One. 2021 Jul 21;16(7):e0254001. doi: 10.1371/journal.pone.0254001 (PMC8294501; doi:10.1371/journal.pone.0254001)
Supplement: S2 Table — (DOCX) [file pone.0254001.s002.docx]

**S2 Table.** Weighted Pearson correlations (weighted by the log 10 of the county population) between all of the public health-related variables we collected with the percentage of voters in the county that voted for Donald Trump or Hillary Clinton, and the Republican margin shift (from 2012 to 2016).

|  | **All States** | | **All States** | **All States** | **Battle States** | **Battle States** | **Battle States** | **Flip States** | **Flip States** | **Flip States** |  |
| --- | --- | --- | --- | --- | --- | --- | --- | --- | --- | --- | --- |
| **Variable** | | % Trump 2016 | % Clinton 2016 | Rep. margin change | % Trump 2016 | % Clinton 2016 | Rep. margin change | % Trump 2016 | % Clinton 2016 | Rep. margin change | category |
| Graduation Rate | | 0.35 | -0.34 | 0.14 | 0.29 | -0.31 | 0.11 | 0.17 | -0.18 | -0.02 | Social, Physical and Economic Environment |
| % Some College | | -0.31 | 0.24 | -0.28 | -0.22 | 0.15 | -0.26 | -0.60 | 0.57 | -0.59 | Social, Physical and Economic Environment |
| % Children in Poverty | | 0.01 | 0.08 | 0.14 | -0.02 | 0.10 | 0.03 | 0.14 | -0.10 | 0.52 | Social, Physical and Economic Environment |
| % Single-Parent Households | | -0.35 | 0.43 | 0.03 | -0.39 | 0.45 | -0.07 | -0.21 | 0.25 | 0.24 | Social, Physical and Economic Environment |
| Violent Crime Rate | | -0.32 | 0.36 | -0.22 | -0.32 | 0.38 | -0.29 | -0.32 | 0.35 | -0.09 | Social, Physical and Economic Environment |
| Injury Death Rate | | 0.30 | -0.29 | 0.30 | 0.18 | -0.16 | 0.21 | 0.11 | -0.07 | 0.34 | Social, Physical and Economic Environment |
| % Severe Housing Problems | | -0.59 | 0.60 | -0.36 | -0.56 | 0.61 | -0.37 | -0.57 | 0.58 | -0.17 | Social, Physical and Economic Environment |
| % Disconnected Youth | | 0.25 | -0.20 | 0.24 | 0.31 | -0.26 | 0.25 | 0.25 | -0.23 | 0.48 | Social, Physical and Economic Environment |
| Household Income | | -0.27 | 0.22 | -0.37 | -0.21 | 0.15 | -0.33 | -0.31 | 0.29 | -0.68 | Social, Physical and Economic Environment |
| Firearm Fatalities Rate | | 0.26 | -0.23 | 0.15 | 0.22 | -0.17 | 0.15 | 0.16 | -0.13 | 0.41 | Social, Physical and Economic Environment |
| % Homeowners | | 0.50 | -0.50 | 0.36 | 0.47 | -0.48 | 0.51 | 0.58 | -0.59 | 0.43 | Social, Physical and Economic Environment |
| % Severe Housing Cost Burden | | -0.60 | 0.62 | -0.32 | -0.55 | 0.62 | -0.37 | -0.52 | 0.54 | -0.22 | Social, Physical and Economic Environment |
| Asbestosis | | 0.03 | -0.03 | -0.04 | -0.06 | 0.08 | 0.02 | -0.06 | 0.03 | 0.11 | Respiratory diseases |
| Asthma | | -0.20 | 0.24 | -0.04 | 0.01 | 0.01 | 0.02 | -0.25 | 0.27 | 0.17 | Respiratory diseases |
| Chronic obstructive pulmonary | | 0.50 | -0.45 | 0.29 | 0.36 | -0.33 | 0.15 | 0.19 | -0.21 | 0.50 | Respiratory diseases |
| Chronic respiratory diseases | | 0.47 | -0.42 | 0.28 | 0.32 | -0.29 | 0.14 | 0.16 | -0.17 | 0.48 | Respiratory diseases |
| Coal workers pneumoconiosis | | 0.11 | -0.10 | 0.07 | 0.10 | -0.07 | 0.13 | 0.23 | -0.19 | 0.18 | Respiratory diseases |
| Interstitial lung disease | | -0.23 | 0.22 | -0.07 | -0.22 | 0.23 | -0.10 | -0.30 | 0.26 | -0.08 | Respiratory diseases |
| Other chronic respiratory | | 0.15 | -0.11 | 0.11 | 0.11 | -0.09 | 0.13 | -0.11 | 0.09 | 0.39 | Respiratory diseases |
| Other pneumoconiosis | | 0.29 | -0.23 | 0.04 | 0.30 | -0.29 | 0.30 | 0.53 | -0.50 | 0.36 | Respiratory diseases |
| Pneumoconiosis | | 0.12 | -0.11 | 0.06 | 0.09 | -0.07 | 0.16 | 0.22 | -0.20 | 0.23 | Respiratory diseases |
| Silicosis | | 0.00 | -0.03 | -0.01 | -0.02 | -0.07 | 0.03 | 0.20 | -0.20 | 0.06 | Respiratory diseases |
| Mortality risk, age 0-5 | | 0.11 | -0.02 | 0.04 | -0.07 | 0.15 | -0.15 | -0.13 | 0.17 | 0.24 | Life expectancy and Mortality |
| Mortality risk, age 25-45 | | 0.15 | -0.06 | 0.11 | 0.00 | 0.07 | -0.03 | 0.03 | 0.02 | 0.46 | Life expectancy and Mortality |
| Mortality risk, age 45-65 | | 0.20 | -0.11 | 0.18 | 0.03 | 0.05 | 0.03 | 0.04 | 0.00 | 0.46 | Life expectancy and Mortality |
| Mortality risk, age 5-25 | | 0.26 | -0.19 | 0.14 | 0.14 | -0.08 | 0.05 | 0.28 | -0.24 | 0.59 | Life expectancy and Mortality |
| Mortality risk, age 65-85 | | 0.30 | -0.22 | 0.23 | 0.04 | 0.02 | 0.15 | 0.16 | -0.14 | 0.45 | Life expectancy and Mortality |
| prct_male_under_18_medicaid | | -0.07 | 0.14 | 0.16 | -0.10 | 0.18 | 0.09 | 0.10 | -0.08 | 0.51 | Insurance and Healthcare cost |
| prct_male_18_64_medicaid | | -0.13 | 0.15 | 0.30 | -0.18 | 0.17 | 0.35 | 0.09 | -0.09 | 0.57 | Insurance and Healthcare cost |
| prct_male_over_64_medicaid | | -0.19 | 0.25 | 0.00 | -0.19 | 0.24 | 0.07 | -0.16 | 0.16 | 0.32 | Insurance and Healthcare cost |
| prct_male_medicaid | | -0.13 | 0.18 | 0.21 | -0.19 | 0.23 | 0.22 | 0.03 | -0.02 | 0.53 | Insurance and Healthcare cost |
| prct_female_under_18_medicaid | | -0.06 | 0.13 | 0.16 | -0.09 | 0.16 | 0.10 | 0.11 | -0.10 | 0.50 | Insurance and Healthcare cost |
| prct_female_18_64_medicaid | | -0.13 | 0.16 | 0.33 | -0.18 | 0.17 | 0.37 | 0.08 | -0.08 | 0.55 | Insurance and Healthcare cost |
| prct_female_over_64_medicaid | | -0.20 | 0.27 | 0.01 | -0.23 | 0.29 | -0.01 | -0.15 | 0.14 | 0.31 | Insurance and Healthcare cost |
| prct_female_medicaid | | -0.12 | 0.18 | 0.23 | -0.18 | 0.21 | 0.24 | 0.04 | -0.03 | 0.53 | Insurance and Healthcare cost |
| prcnt_no_highs_25_64_with_ins | | -0.04 | 0.02 | 0.33 | -0.01 | -0.06 | 0.34 | -0.02 | 0.02 | 0.08 | Insurance and Healthcare cost |
| prcnt_no_highs_25_64_with_private_ins | | 0.11 | -0.15 | -0.02 | 0.23 | -0.28 | 0.06 | 0.11 | -0.11 | -0.24 | Insurance and Healthcare cost |
| prcnt_no_highs_25_64_with_public_ins | | -0.13 | 0.15 | 0.35 | -0.23 | 0.21 | 0.30 | -0.09 | 0.09 | 0.30 | Insurance and Healthcare cost |
| prcnt_yes_highs_25_64_with_ins | | 0.01 | -0.05 | 0.32 | 0.06 | -0.13 | 0.40 | 0.20 | -0.20 | -0.08 | Insurance and Healthcare cost |
| prcnt_yes_highs_25_64_with_private_ins | | 0.18 | -0.22 | 0.10 | 0.25 | -0.30 | 0.16 | 0.21 | -0.21 | -0.26 | Insurance and Healthcare cost |
| prcnt_yes_highs_25_64_with_public_ins | | -0.22 | 0.23 | 0.22 | -0.30 | 0.29 | 0.26 | -0.12 | 0.11 | 0.38 | Insurance and Healthcare cost |
| prcnt_bachelor_25_64_with_ins | | 0.01 | -0.04 | 0.08 | 0.04 | -0.11 | 0.16 | -0.06 | 0.07 | -0.27 | Insurance and Healthcare cost |
| prcnt_bachelor_25_64_with_private_ins | | 0.08 | -0.10 | -0.06 | 0.08 | -0.12 | -0.06 | -0.13 | 0.14 | -0.43 | Insurance and Healthcare cost |
| prcnt_bachelor_25_64_with_public_ins | | -0.08 | 0.09 | 0.20 | -0.04 | 0.05 | 0.28 | 0.22 | -0.23 | 0.50 | Insurance and Healthcare cost |
| wnh_prcnt_18_dis | | 0.13 | -0.11 | 0.17 | 0.07 | -0.03 | 0.19 | 0.32 | -0.31 | 0.34 | Insurance and Healthcare cost |
| wnh_prcnt_18_64_dis | | 0.30 | -0.25 | 0.29 | 0.15 | -0.10 | 0.24 | 0.39 | -0.38 | 0.64 | Insurance and Healthcare cost |
| wnh_prcnt_65_dis | | 0.33 | -0.28 | 0.17 | 0.23 | -0.18 | 0.12 | 0.32 | -0.30 | 0.45 | Insurance and Healthcare cost |
| w_prcnt_18_dis | | 0.12 | -0.09 | 0.18 | 0.08 | -0.04 | 0.21 | 0.30 | -0.29 | 0.33 | Insurance and Healthcare cost |
| w_prcnt_18_64_dis | | 0.31 | -0.26 | 0.32 | 0.17 | -0.11 | 0.27 | 0.38 | -0.38 | 0.63 | Insurance and Healthcare cost |
| w_prcnt_65_dis | | 0.30 | -0.25 | 0.15 | 0.21 | -0.17 | 0.10 | 0.32 | -0.29 | 0.45 | Insurance and Healthcare cost |
| Uninsured %: <= 138% of Poverty | | 0.24 | -0.20 | -0.36 | 0.14 | -0.06 | -0.43 | -0.13 | 0.10 | -0.13 | Insurance and Healthcare cost |
| Uninsured %: <= 400% of Poverty | | 0.18 | -0.13 | -0.33 | 0.05 | 0.03 | -0.40 | -0.13 | 0.11 | 0.03 | Insurance and Healthcare cost |
| Uninsured %: All Incomes | | 0.22 | -0.16 | -0.21 | 0.10 | -0.02 | -0.27 | 0.03 | -0.04 | 0.31 | Insurance and Healthcare cost |
| Part B Drugs Actual Costs | | -0.34 | 0.35 | -0.31 | -0.30 | 0.33 | -0.39 | -0.47 | 0.51 | -0.48 | Insurance and Healthcare cost |
| Emergency Department Visits | | -0.41 | 0.42 | -0.31 | -0.42 | 0.46 | -0.40 | -0.51 | 0.55 | -0.39 | Insurance and Healthcare cost |
| Imaging Per Capita Actual Costs | | -0.18 | 0.24 | -0.30 | -0.13 | 0.19 | -0.40 | -0.09 | 0.14 | -0.22 | Insurance and Healthcare cost |
| Procedures Per Capita Actual Costs | | -0.23 | 0.27 | -0.46 | -0.08 | 0.15 | -0.46 | -0.05 | 0.11 | -0.35 | Insurance and Healthcare cost |
| Hospice Per Capita Actual Costs | | -0.03 | 0.06 | -0.28 | -0.16 | 0.18 | -0.37 | -0.22 | 0.20 | -0.34 | Insurance and Healthcare cost |
| Tests Per Capita Actual Costs | | -0.10 | 0.18 | -0.30 | -0.13 | 0.21 | -0.45 | -0.18 | 0.23 | -0.27 | Insurance and Healthcare cost |
| Actual Per Capita Costs | | -0.11 | 0.18 | -0.13 | -0.04 | 0.10 | -0.13 | -0.16 | 0.22 | -0.18 | Insurance and Healthcare cost |
| Percent Eligible for Medicaid | | -0.17 | 0.23 | 0.18 | -0.27 | 0.30 | 0.22 | -0.13 | 0.11 | 0.34 | Insurance and Healthcare cost |
| Percent Male | | 0.21 | -0.24 | 0.22 | 0.17 | -0.21 | 0.41 | 0.35 | -0.36 | 0.53 | Insurance and Healthcare cost |
| Percent Female | | -0.21 | 0.24 | -0.22 | -0.17 | 0.21 | -0.41 | -0.35 | 0.36 | -0.53 | Insurance and Healthcare cost |
| Diarrheal diseases | | -0.10 | 0.12 | 0.11 | -0.20 | 0.25 | -0.27 | 0.11 | -0.08 | -0.22 | Infectious diseases |
| Hepatitis | | -0.11 | 0.13 | -0.26 | -0.03 | 0.06 | -0.18 | -0.38 | 0.41 | -0.18 | Infectious diseases |
| HIV AIDS | | -0.32 | 0.39 | -0.21 | -0.12 | 0.18 | -0.21 | -0.43 | 0.49 | -0.26 | Infectious diseases |
| Lower respiratory infections | | 0.16 | -0.08 | 0.06 | 0.02 | 0.05 | -0.08 | 0.07 | -0.01 | -0.02 | Infectious diseases |
| Meningitis | | -0.15 | 0.24 | -0.15 | -0.25 | 0.31 | -0.33 | -0.30 | 0.37 | -0.09 | Infectious diseases |
| Tuberculosis | | -0.35 | 0.42 | -0.25 | -0.33 | 0.41 | -0.38 | -0.47 | 0.52 | -0.22 | Infectious diseases |
| Years of Potential Life Lost Rate | | 0.21 | -0.14 | 0.24 | 0.08 | -0.01 | 0.09 | 0.15 | -0.09 | 0.40 | Health Outcomes |
| YPLL Rate (Black) | | 0.08 | -0.07 | 0.09 | -0.02 | 0.05 | 0.33 | 0.00 | 0.01 | 0.33 | Health Outcomes |
| YPLL Rate (White) | | 0.37 | -0.30 | 0.42 | 0.42 | -0.34 | 0.40 | 0.29 | -0.23 | 0.67 | Health Outcomes |
| % Fair/Poor | | -0.01 | 0.10 | 0.04 | -0.12 | 0.19 | -0.13 | -0.03 | 0.06 | 0.36 | Health Outcomes |
| Physically Unhealthy Days | | 0.09 | -0.02 | 0.15 | -0.14 | 0.20 | -0.04 | 0.07 | -0.05 | 0.38 | Health Outcomes |
| Mentally Unhealthy Days | | 0.06 | -0.01 | 0.13 | -0.19 | 0.27 | -0.10 | 0.11 | -0.07 | 0.26 | Health Outcomes |
| % LBW | | -0.14 | 0.23 | -0.09 | -0.23 | 0.33 | -0.28 | -0.21 | 0.26 | -0.06 | Health Outcomes |
| Life Expectancy | | -0.27 | 0.20 | -0.26 | -0.11 | 0.04 | -0.16 | -0.19 | 0.15 | -0.47 | Health Outcomes |
| Life Expectancy (Black) | | -0.16 | 0.11 | -0.21 | -0.02 | -0.03 | -0.16 | 0.01 | -0.04 | -0.17 | Health Outcomes |
| Life Expectancy (White) | | -0.44 | 0.36 | -0.40 | -0.37 | 0.29 | -0.40 | -0.27 | 0.22 | -0.71 | Health Outcomes |
| Age-Adjusted Mortality | | 0.24 | -0.16 | 0.24 | 0.11 | -0.04 | 0.11 | 0.16 | -0.11 | 0.46 | Health Outcomes |
| Age-Adjusted Mortality (Black) | | 0.06 | -0.06 | 0.05 | 0.10 | -0.07 | 0.39 | 0.22 | -0.21 | 0.43 | Health Outcomes |
| Age-Adjusted Mortality (White) | | 0.39 | -0.32 | 0.44 | 0.42 | -0.35 | 0.43 | 0.24 | -0.19 | 0.72 | Health Outcomes |
| Child Mortality Rate | | 0.15 | -0.10 | 0.17 | 0.02 | 0.03 | 0.07 | 0.04 | -0.01 | 0.22 | Health Outcomes |
| Infant Mortality Rate | | 0.17 | -0.11 | 0.29 | 0.01 | 0.06 | 0.16 | -0.07 | 0.10 | 0.31 | Health Outcomes |
| % Frequent Physical Distress | | 0.04 | 0.03 | 0.13 | -0.15 | 0.22 | -0.07 | 0.03 | -0.01 | 0.38 | Health Outcomes |
| % Frequent Mental Distress | | 0.04 | 0.02 | 0.15 | -0.18 | 0.26 | -0.08 | 0.05 | -0.02 | 0.36 | Health Outcomes |
| HIV Prevalence Rate | | -0.43 | 0.49 | -0.25 | -0.24 | 0.33 | -0.33 | -0.47 | 0.52 | -0.27 | Health Outcomes |
| diabetes_crude | | 0.21 | -0.14 | 0.25 | 0.18 | -0.12 | 0.19 | 0.39 | -0.37 | 0.39 | Health Behaviors |
| obesity_crude | | 0.21 | -0.16 | 0.30 | 0.24 | -0.22 | 0.36 | 0.30 | -0.30 | 0.41 | Health Behaviors |
| physical_inactivity_crude | | 0.40 | -0.31 | 0.33 | 0.39 | -0.32 | 0.24 | 0.47 | -0.44 | 0.55 | Health Behaviors |
| % Smokers | | 0.18 | -0.10 | 0.38 | 0.02 | 0.04 | 0.18 | 0.06 | -0.06 | 0.45 | Health Behaviors |
| Food Environment Index | | 0.05 | -0.09 | 0.04 | 0.04 | -0.07 | 0.15 | 0.19 | -0.22 | -0.18 | Health Behaviors |
| % Excessive Drinking | | -0.19 | 0.14 | 0.04 | -0.13 | 0.06 | 0.16 | -0.33 | 0.30 | -0.26 | Health Behaviors |
| Teen Birth Rate | | 0.25 | -0.18 | 0.13 | 0.14 | -0.10 | 0.02 | 0.19 | -0.16 | 0.43 | Health Behaviors |
| % Food Insecure | | -0.11 | 0.18 | -0.05 | -0.21 | 0.28 | -0.21 | -0.18 | 0.21 | 0.19 | Health Behaviors |
| Drug Overdose Mortality Rate | | 0.14 | -0.12 | 0.30 | 0.10 | -0.06 | 0.11 | -0.01 | 0.09 | 0.04 | Health Behaviors |
| MV Mortality Rate | | 0.40 | -0.35 | 0.24 | 0.33 | -0.30 | 0.24 | 0.41 | -0.39 | 0.56 | Health Behaviors |
| % Insufficient Sleep | | -0.13 | 0.23 | 0.04 | -0.23 | 0.32 | -0.03 | 0.07 | -0.02 | 0.14 | Health Behaviors |
| opioid_prescribing_rate | | 0.16 | -0.13 | 0.00 | 0.12 | -0.07 | -0.04 | 0.12 | -0.12 | 0.18 | Health Behaviors |
| % 65 and over | | 0.30 | -0.28 | 0.37 | 0.36 | -0.32 | 0.28 | 0.40 | -0.39 | 0.42 | Demographic |
| % Non-Hispanic White | | 0.54 | -0.59 | 0.45 | 0.52 | -0.56 | 0.50 | 0.61 | -0.66 | 0.38 | Demographic |
| % Rural | | 0.52 | -0.49 | 0.47 | 0.48 | -0.48 | 0.53 | 0.48 | -0.50 | 0.60 | Demographic |
| Alcohol use disorders | | -0.22 | 0.17 | -0.03 | -0.27 | 0.25 | -0.03 | -0.39 | 0.38 | 0.08 | Deaths of Despair |
| Drug use disorders | | 0.12 | -0.10 | 0.10 | -0.12 | 0.17 | -0.10 | -0.10 | 0.16 | 0.03 | Deaths of Despair |
| Interpersonal violence | | -0.27 | 0.35 | -0.13 | -0.29 | 0.36 | -0.30 | -0.39 | 0.45 | -0.10 | Deaths of Despair |
| Self-harm | | 0.26 | -0.30 | 0.15 | 0.16 | -0.16 | 0.18 | 0.21 | -0.19 | 0.58 | Deaths of Despair |
| % With Access | | -0.40 | 0.35 | -0.29 | -0.41 | 0.39 | -0.26 | -0.34 | 0.34 | -0.42 | Clinical Care |
| PCP Rate | | -0.38 | 0.34 | -0.30 | -0.33 | 0.30 | -0.24 | -0.37 | 0.36 | -0.40 | Clinical Care |
| Dentist Rate | | -0.41 | 0.37 | -0.27 | -0.37 | 0.34 | -0.27 | -0.49 | 0.50 | -0.44 | Clinical Care |
| MHP Rate | | -0.42 | 0.38 | -0.24 | -0.48 | 0.47 | -0.28 | -0.54 | 0.51 | -0.36 | Clinical Care |
| Preventable Hosp. Rate | | 0.15 | -0.08 | 0.16 | -0.03 | 0.07 | 0.15 | -0.04 | 0.08 | 0.15 | Clinical Care |
| % Screened | | -0.17 | 0.16 | 0.07 | -0.07 | 0.06 | 0.12 | 0.00 | -0.04 | -0.10 | Clinical Care |
| % Vaccinated | | -0.23 | 0.21 | -0.12 | -0.33 | 0.34 | -0.27 | -0.35 | 0.35 | -0.53 | Clinical Care |
| Aortic aneurysm | | 0.31 | -0.32 | 0.47 | 0.16 | -0.18 | 0.53 | 0.21 | -0.23 | 0.47 | Cardiovascular diseases |
| Atrial fibrillation & flutter | | 0.07 | -0.13 | 0.00 | 0.05 | -0.08 | 0.16 | -0.04 | 0.02 | 0.05 | Cardiovascular diseases |
| Cardiomyopathy & myocarditis | | -0.17 | 0.25 | -0.04 | -0.21 | 0.31 | -0.17 | -0.03 | 0.07 | -0.11 | Cardiovascular diseases |
| Cardiovascular diseases | | 0.27 | -0.19 | 0.23 | 0.11 | -0.03 | 0.21 | 0.24 | -0.19 | 0.41 | Cardiovascular diseases |
| Cerebrovascular disease | | 0.16 | -0.10 | 0.06 | -0.03 | 0.08 | 0.02 | -0.02 | 0.03 | 0.07 | Cardiovascular diseases |
| Endocarditis | | 0.07 | -0.08 | 0.06 | 0.13 | -0.10 | 0.07 | 0.07 | -0.04 | -0.02 | Cardiovascular diseases |
| Hemorrhagic stroke | | 0.04 | 0.05 | 0.02 | -0.11 | 0.18 | -0.04 | -0.02 | 0.06 | 0.12 | Cardiovascular diseases |
| Hypertensive heart disease | | -0.12 | 0.17 | -0.13 | -0.19 | 0.23 | -0.22 | -0.34 | 0.36 | -0.13 | Cardiovascular diseases |
| Ischemic heart disease | | 0.32 | -0.24 | 0.29 | 0.18 | -0.10 | 0.27 | 0.32 | -0.27 | 0.47 | Cardiovascular diseases |
| Ischemic stroke | | 0.20 | -0.17 | 0.08 | 0.00 | 0.03 | 0.04 | -0.02 | 0.02 | 0.04 | Cardiovascular diseases |
| Other cardiovascular | | 0.04 | -0.03 | 0.12 | 0.12 | -0.10 | 0.19 | 0.08 | -0.06 | 0.00 | Cardiovascular diseases |
| Peripheral vascular disease | | 0.07 | -0.02 | 0.14 | -0.05 | 0.08 | 0.10 | 0.06 | -0.06 | 0.12 | Cardiovascular diseases |
| Rheumatic heart disease | | 0.17 | -0.15 | 0.00 | 0.27 | -0.30 | 0.31 | 0.15 | -0.14 | 0.31 | Cardiovascular diseases |
| Acute lymphoid leukemia | | 0.16 | -0.09 | -0.01 | 0.07 | -0.06 | 0.05 | 0.26 | -0.22 | 0.46 | Cancers |
| Acute myeloid leukemia | | 0.30 | -0.28 | 0.42 | 0.17 | -0.18 | 0.49 | 0.17 | -0.18 | 0.41 | Cancers |
| Bladder cancer | | 0.13 | -0.13 | 0.35 | 0.03 | -0.01 | 0.26 | 0.19 | -0.21 | 0.31 | Cancers |
| Brain & nervous system cancer | | 0.50 | -0.50 | 0.31 | 0.34 | -0.38 | 0.37 | 0.10 | -0.17 | 0.36 | Cancers |
| Breast cancer | | -0.01 | 0.09 | -0.01 | -0.11 | 0.17 | -0.11 | -0.04 | 0.10 | 0.08 | Cancers |
| Cervical cancer | | -0.01 | 0.11 | 0.08 | -0.12 | 0.21 | -0.08 | 0.06 | 0.01 | 0.26 | Cancers |
| Chronic lymphoid leukemia | | 0.41 | -0.41 | 0.51 | 0.27 | -0.32 | 0.54 | 0.37 | -0.38 | 0.34 | Cancers |
| Chronic myeloid leukemia | | 0.26 | -0.21 | 0.20 | 0.00 | 0.00 | 0.24 | 0.08 | -0.09 | 0.32 | Cancers |
| Colon & rectum cancer | | 0.23 | -0.15 | 0.34 | 0.23 | -0.19 | 0.25 | 0.23 | -0.18 | 0.40 | Cancers |
| Esophageal cancer | | 0.01 | 0.00 | 0.44 | 0.04 | -0.06 | 0.43 | 0.21 | -0.22 | 0.49 | Cancers |
| Gallbladder & biliary tract | | -0.14 | 0.14 | 0.25 | -0.15 | 0.10 | 0.27 | -0.08 | 0.12 | 0.18 | Cancers |
| Hodgkin lymphoma | | 0.08 | 0.00 | 0.19 | 0.15 | -0.14 | 0.36 | 0.26 | -0.25 | 0.40 | Cancers |
| Kidney cancer | | 0.39 | -0.35 | 0.37 | 0.17 | -0.20 | 0.39 | 0.13 | -0.13 | 0.49 | Cancers |
| Larynx cancer | | 0.03 | 0.08 | 0.20 | -0.08 | 0.18 | -0.06 | -0.04 | 0.07 | 0.33 | Cancers |
| Leukemia | | 0.38 | -0.35 | 0.46 | 0.22 | -0.24 | 0.52 | 0.28 | -0.28 | 0.44 | Cancers |
| Lip & oral cavity cancer | | 0.08 | 0.01 | 0.03 | 0.00 | 0.07 | -0.03 | -0.02 | 0.00 | 0.45 | Cancers |
| Liver cancer | | -0.17 | 0.24 | -0.13 | -0.26 | 0.31 | -0.14 | -0.29 | 0.35 | 0.21 | Cancers |
| Malignant skin melanoma | | 0.56 | -0.58 | 0.17 | 0.48 | -0.45 | -0.03 | 0.35 | -0.37 | 0.15 | Cancers |
| Mesothelioma | | -0.05 | 0.01 | 0.13 | -0.12 | 0.09 | 0.27 | -0.07 | 0.05 | 0.02 | Cancers |
| Multiple myeloma | | -0.18 | 0.23 | 0.00 | -0.25 | 0.27 | 0.03 | -0.23 | 0.24 | 0.08 | Cancers |
| Nasopharynx cancer | | -0.23 | 0.32 | -0.11 | -0.23 | 0.32 | -0.22 | -0.32 | 0.35 | 0.15 | Cancers |
| Neoplasms | | 0.22 | -0.13 | 0.32 | 0.04 | 0.01 | 0.22 | 0.08 | -0.05 | 0.49 | Cancers |
| Non-Hodgkin lymphoma | | 0.32 | -0.32 | 0.44 | 0.16 | -0.19 | 0.41 | 0.38 | -0.38 | 0.25 | Cancers |
| Non-melanoma skin cancer | | 0.35 | -0.33 | 0.04 | 0.15 | -0.10 | -0.19 | 0.17 | -0.19 | 0.30 | Cancers |
| Other neoplasms | | 0.07 | -0.01 | 0.30 | -0.08 | 0.07 | 0.39 | -0.04 | 0.03 | 0.36 | Cancers |
| Other pharynx cancer | | -0.11 | 0.21 | 0.07 | -0.17 | 0.25 | -0.05 | -0.16 | 0.16 | 0.32 | Cancers |
| Ovarian cancer | | 0.04 | -0.05 | 0.08 | 0.08 | -0.07 | 0.22 | 0.18 | -0.14 | 0.14 | Cancers |
| Pancreatic cancer | | -0.09 | 0.16 | 0.13 | -0.21 | 0.25 | 0.16 | -0.14 | 0.18 | 0.14 | Cancers |
| Prostate cancer | | -0.19 | 0.23 | -0.03 | -0.21 | 0.20 | 0.13 | -0.20 | 0.21 | 0.07 | Cancers |
| Stomach cancer | | -0.34 | 0.43 | -0.16 | -0.44 | 0.49 | -0.14 | -0.31 | 0.38 | 0.02 | Cancers |
| Testicular cancer | | 0.32 | -0.30 | 0.29 | 0.28 | -0.27 | 0.23 | 0.43 | -0.42 | 0.54 | Cancers |
| Thyroid cancer | | -0.10 | 0.10 | 0.00 | -0.14 | 0.09 | 0.18 | -0.07 | 0.12 | 0.12 | Cancers |
| Tracheal, bronchus, & lung | | 0.32 | -0.23 | 0.35 | 0.10 | -0.03 | 0.18 | 0.11 | -0.09 | 0.52 | Cancers |
| Uterine cancer | | -0.34 | 0.36 | 0.21 | -0.25 | 0.27 | 0.12 | -0.22 | 0.26 | -0.06 | Cancers |
