# Supplementary material for: Viewing the US presidential electoral map through the lens of public health
Source: PLoS One. 2021 Jul 21;16(7):e0254001. doi: 10.1371/journal.pone.0254001 (PMC8294501; doi:10.1371/journal.pone.0254001)
Supplement: S3 Table — Every county was assigned as either Republican or Democratic depending on the majority vote in 2016, and the mean, median, 1st quartile, and 3rd quartile values for different public health-related variables were calculated. The differences in these values for Republican and Democratic counties are presented in S3 Table, along with the Student t-test statistics and p values for the mean comparisons. (DOCX) [file pone.0254001.s003.docx]

**S3 Table.** Quantile and mean comparisons of Republican and Democratic counties across all of the public-health measures we collected. Every county was assigned as either Republican or Democratic depending on the majority vote in 2016, and the mean, median, 1st quartile, and 3rd quartile values for different public health-related variables were calculated. The differences in these values for Republican and Democratic counties are presented in Supplementary Table 3, along with the Student t-test statistics and p values for the mean comparisons.

| key | % Mean Difference | % Median Difference | % Top Quartile Difference | % Bottom Quartile Difference | t-test p value | t-test t statistic | category |
| --- | --- | --- | --- | --- | --- | --- | --- |
| Violent Crime Rate | -39% | -41% | -39% | -36% | 2.1E-27 | -1.1E+01 | Social, Physical and Economic Environment |
| % Severe Housing Cost Burden | -32% | -33% | -31% | -35% | 3.7E-110 | -2.8E+01 | Social, Physical and Economic Environment |
| % Severe Housing Problems | -30% | -29% | -29% | -31% | 9.1E-106 | -2.7E+01 | Social, Physical and Economic Environment |
| % Single-Parent Households | -22% | -16% | -25% | -13% | 2.9E-34 | -1.3E+01 | Social, Physical and Economic Environment |
| % Children in Poverty | -10% | 1% | -22% | 18% | 1.1E-04 | -3.9E+00 | Social, Physical and Economic Environment |
| Household Income | -10% | -10% | -16% | 9% | 8.9E-09 | -5.8E+00 | Social, Physical and Economic Environment |
| % Some College | -9% | -11% | -11% | -6% | 5.7E-15 | -8.0E+00 | Social, Physical and Economic Environment |
| Graduation Rate | 5% | 5% | 5% | 5% | 2.5E-30 | 1.2E+01 | Social, Physical and Economic Environment |
| % Homeowners | 15% | 14% | 10% | 20% | 3.2E-68 | 2.0E+01 | Social, Physical and Economic Environment |
| Firearm Fatalities Rate | 16% | 29% | 5% | 51% | 3.9E-07 | 5.1E+00 | Social, Physical and Economic Environment |
| Injury Death Rate | 17% | 17% | 16% | 26% | 2.9E-19 | 9.3E+00 | Social, Physical and Economic Environment |
| % Disconnected Youth | 20% | 26% | 20% | 26% | 7.1E-06 | 4.5E+00 | Social, Physical and Economic Environment |
| Asthma | -16% | -2% | -24% | 6% | 1.8E-12 | -7.2E+00 | Respiratory diseases |
| Silicosis | -8% | 0% | 33% | 0% | 5.6E-01 | -5.9E-01 | Respiratory diseases |
| Interstitial lung disease | -8% | -7% | -7% | -6% | 6.3E-11 | -6.7E+00 | Respiratory diseases |
| Other chronic respiratory | 2% | 10% | -6% | 18% | 1.1E-01 | 1.6E+00 | Respiratory diseases |
| Asbestosis | 3% | -7% | 4% | -10% | 6.1E-01 | 5.1E-01 | Respiratory diseases |
| Other pneumoconiosis | 10% | 26% | 8% | 25% | 1.4E-05 | 4.4E+00 | Respiratory diseases |
| Chronic respiratory diseases | 27% | 25% | 24% | 27% | 1.9E-70 | 2.0E+01 | Respiratory diseases |
| Chronic obstructive pulmonary | 33% | 32% | 31% | 33% | 2.0E-85 | 2.2E+01 | Respiratory diseases |
| Pneumoconiosis | 37% | 9% | 8% | 15% | 3.1E-09 | 5.9E+00 | Respiratory diseases |
| Coal workers pneumoconiosis | 360% | 50% | 75% | 0% | 8.0E-10 | 6.2E+00 | Respiratory diseases |
| Mortality risk, age 0-5 | -2% | 11% | -11% | 17% | 2.5E-01 | -1.1E+00 | Life expectancy and Mortality |
| Mortality risk, age 25-45 | 1% | 11% | -8% | 19% | 7.7E-01 | 2.9E-01 | Life expectancy and Mortality |
| Mortality risk, age 45-65 | 4% | 9% | -2% | 13% | 1.2E-02 | 2.5E+00 | Life expectancy and Mortality |
| Mortality risk, age 65-85 | 5% | 6% | 3% | 7% | 3.0E-13 | 7.5E+00 | Life expectancy and Mortality |
| Mortality risk, age 5-25 | 9% | 25% | 0% | 32% | 4.3E-05 | 4.1E+00 | Life expectancy and Mortality |
| Part B Drugs Actual Costs | -97% | -86% | -91% | -60% | 2.7E-03 | -3.0E+00 | Insurance and Healthcare cost |
| Emergency Department Visits | -97% | -82% | -90% | -65% | 2.7E-03 | -3.0E+00 | Insurance and Healthcare cost |
| prct_male_over_64_medicaid | -24% | -16% | -26% | -15% | 3.7E-19 | -9.3E+00 | Insurance and Healthcare cost |
| prct_female_over_64_medicaid | -24% | -17% | -28% | -12% | 4.9E-20 | -9.5E+00 | Insurance and Healthcare cost |
| Percent Eligible for Medicaid | -15% | -9% | -16% | -12% | 1.5E-14 | -7.9E+00 | Insurance and Healthcare cost |
| Tests Per Capita Actual Costs | -14% | -17% | -12% | -19% | 5.7E-15 | -8.0E+00 | Insurance and Healthcare cost |
| prcnt_yes_highs_25_64_with_public_ins | -14% | -12% | -13% | -17% | 2.7E-15 | -8.1E+00 | Insurance and Healthcare cost |
| Imaging Per Capita Actual Costs | -13% | -14% | -14% | -11% | 5.0E-16 | -8.3E+00 | Insurance and Healthcare cost |
| prct_male_18_64_medicaid | -13% | -12% | -13% | -15% | 2.7E-07 | -5.2E+00 | Insurance and Healthcare cost |
| prct_male_medicaid | -13% | -10% | -16% | -5% | 2.8E-10 | -6.4E+00 | Insurance and Healthcare cost |
| prct_female_medicaid | -12% | -8% | -16% | -5% | 2.5E-09 | -6.1E+00 | Insurance and Healthcare cost |
| Procedures Per Capita Actual Costs | -11% | -10% | -12% | -10% | 3.6E-26 | -1.1E+01 | Insurance and Healthcare cost |
| prct_female_18_64_medicaid | -11% | -9% | -12% | -12% | 8.2E-07 | -5.0E+00 | Insurance and Healthcare cost |
| prct_male_under_18_medicaid | -9% | -5% | -11% | -4% | 1.6E-06 | -4.8E+00 | Insurance and Healthcare cost |
| prct_female_under_18_medicaid | -9% | -7% | -11% | -4% | 2.9E-06 | -4.7E+00 | Insurance and Healthcare cost |
| prcnt_no_highs_25_64_with_public_ins | -9% | -8% | -7% | -13% | 6.9E-06 | -4.5E+00 | Insurance and Healthcare cost |
| prcnt_bachelor_25_64_with_public_ins | -8% | -4% | -9% | -7% | 2.8E-03 | -3.0E+00 | Insurance and Healthcare cost |
| Hospice Per Capita Actual Costs | -6% | -9% | 1% | -22% | 4.5E-04 | -3.5E+00 | Insurance and Healthcare cost |
| Actual Per Capita Costs | -5% | -4% | -7% | -3% | 1.8E-10 | -6.5E+00 | Insurance and Healthcare cost |
| Percent Female | -2% | -2% | -2% | -1% | 4.3E-20 | -9.4E+00 | Insurance and Healthcare cost |
| prcnt_bachelor_25_64_with_ins | 1% | 1% | 0% | 1% | 3.6E-05 | 4.2E+00 | Insurance and Healthcare cost |
| prcnt_bachelor_25_64_with_private_ins | 2% | 2% | 1% | 2% | 2.4E-08 | 5.7E+00 | Insurance and Healthcare cost |
| prcnt_no_highs_25_64_with_ins | 2% | 3% | 2% | 4% | 3.4E-02 | 2.1E+00 | Insurance and Healthcare cost |
| Percent Male | 2% | 2% | 2% | 2% | 4.3E-20 | 9.4E+00 | Insurance and Healthcare cost |
| Uninsured %: <= 400% of Poverty | 3% | -2% | 3% | -2% | 1.7E-01 | 1.4E+00 | Insurance and Healthcare cost |
| prcnt_yes_highs_25_64_with_ins | 3% | 3% | 2% | 4% | 1.2E-08 | 5.8E+00 | Insurance and Healthcare cost |
| Uninsured %: All Incomes | 6% | 2% | 4% | 11% | 5.9E-03 | 2.8E+00 | Insurance and Healthcare cost |
| w_prcnt_65_dis | 7% | 9% | 8% | 8% | 1.4E-11 | 6.9E+00 | Insurance and Healthcare cost |
| Uninsured %: <= 138% of Poverty | 9% | 11% | 12% | 0% | 3.2E-06 | 4.7E+00 | Insurance and Healthcare cost |
| wnh_prcnt_65_dis | 10% | 10% | 10% | 9% | 1.9E-19 | 9.3E+00 | Insurance and Healthcare cost |
| prcnt_yes_highs_25_64_with_private_ins | 10% | 8% | 9% | 9% | 2.1E-29 | 1.2E+01 | Insurance and Healthcare cost |
| w_prcnt_18_dis | 12% | 18% | 17% | 12% | 1.3E-04 | 3.9E+00 | Insurance and Healthcare cost |
| prcnt_no_highs_25_64_with_private_ins | 13% | 10% | 11% | 10% | 1.7E-16 | 8.4E+00 | Insurance and Healthcare cost |
| wnh_prcnt_18_dis | 16% | 20% | 17% | 16% | 4.2E-07 | 5.1E+00 | Insurance and Healthcare cost |
| wnh_prcnt_18_64_dis | 20% | 27% | 25% | 28% | 1.4E-17 | 8.8E+00 | Insurance and Healthcare cost |
| w_prcnt_18_64_dis | 21% | 24% | 22% | 29% | 3.8E-22 | 1.0E+01 | Insurance and Healthcare cost |
| HIV AIDS | -59% | -58% | -68% | -45% | 3.3E-30 | -1.2E+01 | Infectious diseases |
| Tuberculosis | -43% | -41% | -47% | -35% | 1.3E-35 | -1.3E+01 | Infectious diseases |
| Hepatitis | -21% | -22% | -16% | -24% | 2.1E-14 | -7.8E+00 | Infectious diseases |
| Meningitis | -16% | -7% | -25% | 0% | 2.1E-17 | -8.8E+00 | Infectious diseases |
| Diarrheal diseases | -9% | -13% | -6% | -12% | 2.0E-07 | -5.3E+00 | Infectious diseases |
| Lower respiratory infections | 3% | 2% | -2% | 9% | 1.2E-01 | 1.5E+00 | Infectious diseases |
| HIV Prevalence Rate | -56% | -59% | -58% | -50% | 5.7E-33 | -1.3E+01 | Health Outcomes |
| % LBW | -13% | -9% | -17% | -5% | 5.1E-18 | -8.9E+00 | Health Outcomes |
| % Fair/Poor | -8% | -2% | -14% | 7% | 1.7E-06 | -4.8E+00 | Health Outcomes |
| % Frequent Physical Distress | -3% | 0% | -5% | 3% | 8.5E-03 | -2.6E+00 | Health Outcomes |
| Life Expectancy (White) | -2% | -3% | -3% | -2% | 1.3E-21 | -1.0E+01 | Health Outcomes |
| % Frequent Mental Distress | -2% | 0% | -1% | -1% | 2.2E-02 | -2.3E+00 | Health Outcomes |
| Life Expectancy | -1% | -2% | -2% | -1% | 1.1E-08 | -5.8E+00 | Health Outcomes |
| Life Expectancy (Black) | -1% | -1% | -1% | 0% | 9.9E-03 | -2.6E+00 | Health Outcomes |
| Mentally Unhealthy Days | -1% | 1% | 2% | -3% | 4.0E-01 | -8.4E-01 | Health Outcomes |
| Physically Unhealthy Days | -1% | 1% | 0% | 1% | 5.3E-01 | -6.3E-01 | Health Outcomes |
| Child Mortality Rate | 5% | 13% | 0% | 21% | 5.2E-02 | 1.9E+00 | Health Outcomes |
| Years of Potential Life Lost Rate | 6% | 17% | 1% | 23% | 6.8E-03 | 2.7E+00 | Health Outcomes |
| Infant Mortality Rate | 6% | 13% | 0% | 23% | 1.4E-02 | 2.5E+00 | Health Outcomes |
| Age-Adjusted Mortality | 7% | 16% | 2% | 22% | 1.3E-04 | 3.9E+00 | Health Outcomes |
| YPLL Rate (Black) | 11% | 2% | 2% | 9% | 3.6E-02 | 2.1E+00 | Health Outcomes |
| Age-Adjusted Mortality (White) | 15% | 21% | 12% | 30% | 2.7E-15 | 8.1E+00 | Health Outcomes |
| YPLL Rate (White) | 16% | 22% | 14% | 32% | 2.6E-14 | 7.8E+00 | Health Outcomes |
| Age-Adjusted Mortality (Black) | 18% | 6% | 5% | 12% | 6.4E-02 | 1.9E+00 | Health Outcomes |
| % Food Insecure | -14% | -5% | -20% | 1% | 2.8E-12 | -7.1E+00 | Health Behaviors |
| % Insufficient Sleep | -5% | -5% | -7% | -4% | 4.7E-14 | -7.7E+00 | Health Behaviors |
| % Excessive Drinking | -2% | -4% | -4% | 1% | 3.1E-02 | -2.2E+00 | Health Behaviors |
| % Smokers | 4% | 6% | 1% | 10% | 1.2E-03 | 3.3E+00 | Health Behaviors |
| Food Environment Index | 6% | 3% | 0% | 11% | 4.3E-08 | 5.6E+00 | Health Behaviors |
| obesity_crude | 9% | 13% | 3% | 20% | 2.7E-14 | 7.8E+00 | Health Behaviors |
| Teen Birth Rate | 13% | 31% | 7% | 53% | 5.9E-05 | 4.0E+00 | Health Behaviors |
| diabetes_crude | 14% | 21% | 12% | 20% | 9.1E-13 | 7.3E+00 | Health Behaviors |
| Drug Overdose Mortality Rate | 15% | 13% | 12% | 15% | 6.2E-06 | 4.6E+00 | Health Behaviors |
| opioid_prescribing_rate | 17% | 24% | 26% | 13% | 3.9E-09 | 6.0E+00 | Health Behaviors |
| physical_inactivity_crude | 17% | 19% | 12% | 29% | 2.8E-32 | 1.3E+01 | Health Behaviors |
| MV Mortality Rate | 33% | 69% | 32% | 75% | 2.8E-17 | 8.7E+00 | Health Behaviors |
| % 65 and over | 20% | 21% | 18% | 25% | 1.6E-47 | 1.6E+01 | Demographic |
| % Non-Hispanic White | 50% | 69% | 22% | 102% | 2.7E-85 | 2.3E+01 | Demographic |
| % Rural | 94% | 234% | 81% | 788% | 2.5E-66 | 1.9E+01 | Demographic |
| Interpersonal violence | -39% | -28% | -42% | -9% | 3.1E-23 | -1.0E+01 | Deaths of Despair |
| Alcohol use disorders | -26% | -22% | -19% | -16% | 4.7E-08 | -5.5E+00 | Deaths of Despair |
| Drug use disorders | 9% | 2% | 16% | -4% | 1.4E-03 | 3.2E+00 | Deaths of Despair |
| Self-harm | 18% | 22% | 21% | 23% | 1.2E-17 | 8.8E+00 | Deaths of Despair |
| MHP Rate | -52% | -61% | -51% | -66% | 7.3E-37 | -1.4E+01 | Clinical Care |
| Dentist Rate | -36% | -42% | -34% | -40% | 2.8E-35 | -1.3E+01 | Clinical Care |
| PCP Rate | -34% | -37% | -34% | -33% | 1.0E-32 | -1.3E+01 | Clinical Care |
| % With Access | -19% | -24% | -19% | -25% | 1.3E-27 | -1.1E+01 | Clinical Care |
| % Vaccinated | -6% | -5% | -4% | -8% | 3.0E-08 | -5.6E+00 | Clinical Care |
| % Screened | -1% | -2% | 0% | -3% | 3.1E-01 | -1.0E+00 | Clinical Care |
| Preventable Hosp. Rate | 5% | 6% | 4% | 13% | 2.6E-02 | 2.2E+00 | Clinical Care |
| Hypertensive heart disease | -23% | -18% | -26% | -14% | 2.0E-09 | -6.1E+00 | Cardiovascular diseases |
| Cardiomyopathy & myocarditis | -13% | -15% | -16% | -6% | 2.6E-11 | -6.8E+00 | Cardiovascular diseases |
| Hemorrhagic stroke | -4% | 5% | -8% | 8% | 2.1E-02 | -2.3E+00 | Cardiovascular diseases |
| Peripheral vascular disease | 1% | 4% | -4% | 7% | 4.3E-01 | 7.9E-01 | Cardiovascular diseases |
| Other cardiovascular | 2% | 1% | -1% | 5% | 1.0E-01 | 1.6E+00 | Cardiovascular diseases |
| Endocarditis | 2% | 1% | 0% | 7% | 6.0E-02 | 1.9E+00 | Cardiovascular diseases |
| Rheumatic heart disease | 4% | 10% | 1% | 13% | 2.5E-02 | 2.2E+00 | Cardiovascular diseases |
| Cerebrovascular disease | 4% | 5% | 3% | 8% | 2.0E-03 | 3.1E+00 | Cardiovascular diseases |
| Atrial fibrillation & flutter | 5% | 4% | 3% | 5% | 9.1E-05 | 3.9E+00 | Cardiovascular diseases |
| Cardiovascular diseases | 8% | 11% | 5% | 15% | 3.0E-09 | 6.0E+00 | Cardiovascular diseases |
| Ischemic stroke | 8% | 9% | 7% | 9% | 1.3E-10 | 6.5E+00 | Cardiovascular diseases |
| Aortic aneurysm | 12% | 9% | 8% | 16% | 2.4E-35 | 1.3E+01 | Cardiovascular diseases |
| Ischemic heart disease | 13% | 16% | 13% | 20% | 1.9E-16 | 8.5E+00 | Cardiovascular diseases |
| Nasopharynx cancer | -21% | -10% | -30% | -4% | 1.9E-21 | -9.9E+00 | Cancers |
| Stomach cancer | -20% | -19% | -26% | -13% | 1.8E-42 | -1.5E+01 | Cancers |
| Liver cancer | -13% | -12% | -13% | -10% | 2.7E-20 | -9.6E+00 | Cancers |
| Uterine cancer | -10% | -9% | -13% | -7% | 2.1E-25 | -1.1E+01 | Cancers |
| Prostate cancer | -9% | -3% | -13% | -1% | 2.5E-12 | -7.2E+00 | Cancers |
| Other pharynx cancer | -9% | -3% | -16% | 2% | 4.5E-08 | -5.5E+00 | Cancers |
| Cervical cancer | -8% | 0% | -14% | 7% | 6.8E-06 | -4.5E+00 | Cancers |
| Multiple myeloma | -5% | -1% | -14% | 4% | 1.6E-08 | -5.7E+00 | Cancers |
| Gallbladder & biliary tract | -5% | 1% | -5% | 1% | 1.7E-03 | -3.1E+00 | Cancers |
| Larynx cancer | -4% | 1% | -11% | 6% | 1.8E-02 | -2.4E+00 | Cancers |
| Breast cancer | -4% | 1% | -8% | 3% | 4.1E-04 | -3.6E+00 | Cancers |
| Pancreatic cancer | -3% | -3% | -6% | 1% | 3.1E-06 | -4.7E+00 | Cancers |
| Thyroid cancer | -3% | -2% | -3% | 1% | 8.1E-06 | -4.5E+00 | Cancers |
| Lip & oral cavity cancer | -1% | 3% | -3% | 7% | 3.9E-01 | -8.6E-01 | Cancers |
| Other neoplasms | 1% | 2% | -2% | 5% | 1.9E-01 | 1.3E+00 | Cancers |
| Hodgkin lymphoma | 1% | 3% | -2% | 6% | 9.5E-02 | 1.7E+00 | Cancers |
| Ovarian cancer | 1% | 0% | 1% | 1% | 2.0E-02 | 2.3E+00 | Cancers |
| Mesothelioma | 2% | 8% | -1% | 19% | 4.7E-01 | 7.2E-01 | Cancers |
| Esophageal cancer | 2% | 3% | -2% | 10% | 9.9E-02 | 1.7E+00 | Cancers |
| Acute lymphoid leukemia | 2% | 6% | -1% | 7% | 4.9E-02 | 2.0E+00 | Cancers |
| Chronic myeloid leukemia | 4% | 5% | 2% | 7% | 5.7E-11 | 6.7E+00 | Cancers |
| Colon & rectum cancer | 5% | 10% | 1% | 12% | 2.9E-05 | 4.2E+00 | Cancers |
| Neoplasms | 5% | 7% | 1% | 9% | 7.6E-08 | 5.4E+00 | Cancers |
| Bladder cancer | 6% | 5% | 4% | 7% | 1.1E-12 | 7.3E+00 | Cancers |
| Acute myeloid leukemia | 8% | 7% | 6% | 9% | 4.1E-36 | 1.3E+01 | Cancers |
| Leukemia | 9% | 8% | 7% | 11% | 3.4E-48 | 1.6E+01 | Cancers |
| Non-Hodgkin lymphoma | 10% | 10% | 10% | 9% | 3.1E-57 | 1.8E+01 | Cancers |
| Kidney cancer | 12% | 13% | 9% | 16% | 8.6E-33 | 1.3E+01 | Cancers |
| Non-melanoma skin cancer | 14% | 11% | 13% | 12% | 2.8E-41 | 1.4E+01 | Cancers |
| Chronic lymphoid leukemia | 14% | 12% | 10% | 15% | 7.3E-59 | 1.8E+01 | Cancers |
| Brain & nervous system cancer | 15% | 15% | 12% | 18% | 1.2E-89 | 2.4E+01 | Cancers |
| Testicular cancer | 17% | 22% | 19% | 25% | 9.0E-27 | 1.1E+01 | Cancers |
| Tracheal, bronchus, & lung | 18% | 16% | 14% | 20% | 6.6E-29 | 1.2E+01 | Cancers |
| Malignant skin melanoma | 27% | 27% | 22% | 31% | 9.7E-98 | 2.5E+01 | Cancers |
