# Supplementary material for: Viewing the US presidential electoral map through the lens of public health
Source: PLoS One. 2021 Jul 21;16(7):e0254001. doi: 10.1371/journal.pone.0254001 (PMC8294501; doi:10.1371/journal.pone.0254001)
Supplement: S4 Table — Each linear model included education, socio-economic status, and demographic control variables for the county. (DOCX) [file pone.0254001.s004.docx]

**S4 Table.** This table reports the coefficients for each public health variable under consideration, as well as the standard error for those coefficients, when predicting the percentage of voters in the county that voted for Donald Trump or Hillary Clinton, and the Republican margin shift. Each linear model included education, socio-economic status, and demographic control variables for the county.

| **Variable** | **Rep. margin change** | **% Trump 2016** | **% Clinton 2016** | **category** |
| --- | --- | --- | --- | --- |
| **Gallbladder & biliary tract** | 0.03678 (0.00155) | -0.02317 (0.00204) | 0.02071 (0.00198) | **Cancers** |
| **Uterine cancer** | 0.02993 (0.00141) | -0.03901 (0.00173) | 0.03814 (0.00167) | **Cancers** |
| **Esophageal cancer** | 0.02859 (0.00151) | -0.03156 (0.00189) | 0.03183 (0.00182) | **Cancers** |
| **Non-Hodgkin lymphoma** | 0.02668 (0.0019) | -0.01141 (0.00242) | 0.01404 (0.00233) | **Cancers** |
| **Chronic lymphoid leukemia** | 0.024 (0.00196) | -0.01855 (0.00246) | 0.02182 (0.00237) | **Cancers** |
| **Acute myeloid leukemia** | 0.02248 (0.00166) | -0.0042 (0.00212) | 0.00813 (0.00205) | **Cancers** |
| **Kidney cancer** | 0.02213 (0.00192) | 0.01631 (0.00241) | -0.01153 (0.00234) | **Cancers** |
| **Leukemia** | 0.02191 (0.00176) | -0.00369 (0.00223) | 0.00773 (0.00216) | **Cancers** |
| **Colon & rectum cancer** | 0.01997 (0.00193) | -0.00151 (0.00243) | 0.00984 (0.00234) | **Cancers** |
| **Bladder cancer** | 0.01933 (0.0018) | -0.02045 (0.00224) | 0.02249 (0.00216) | **Cancers** |
| **Other neoplasms** | 0.01875 (0.00168) | -0.02076 (0.00208) | 0.02447 (0.002) | **Cancers** |
| **Stomach cancer** | 0.0164 (0.00288) | -0.02309 (0.00356) | 0.03735 (0.0034) | **Cancers** |
| **Thyroid cancer** | 0.01427 (0.00172) | -0.00427 (0.00215) | -0.00224 (0.00209) | **Cancers** |
| **Neoplasms** | 0.01422 (0.0019) | -0.00072 (0.00237) | 0.01221 (0.00229) | **Cancers** |
| **Larynx cancer** | 0.01247 (0.0019) | -0.01214 (0.00236) | 0.02591 (0.00225) | **Cancers** |
| **Hodgkin lymphoma** | 0.01202 (0.00178) | -0.00787 (0.00222) | 0.01426 (0.00213) | **Cancers** |
| **Tracheal, bronchus, & lung** | 0.01192 (0.00189) | 0.00745 (0.00236) | 0.00634 (0.00228) | **Cancers** |
| **Mesothelioma** | 0.01169 (0.00158) | -0.01756 (0.00194) | 0.0162 (0.00188) | **Cancers** |
| **Cervical cancer** | 0.01142 (0.00246) | -0.00049 (0.00306) | 0.01203 (0.00295) | **Cancers** |
| **Pancreatic cancer** | 0.01142 (0.00167) | -0.01625 (0.00206) | 0.02247 (0.00197) | **Cancers** |
| **Testicular cancer** | 0.00778 (0.00175) | 0.02072 (0.00214) | -0.02245 (0.00206) | **Cancers** |
| **Multiple myeloma** | 0.00357 (0.00163) | -0.02078 (0.00198) | 0.0248 (0.0019) | **Cancers** |
| **Other pharynx cancer** | 0.0031 (0.00184) | -0.01607 (0.00226) | 0.02665 (0.00215) | **Cancers** |
| **Liver cancer** | 0.00126 (0.00204) | 0.00515 (0.00253) | -0.00048 (0.00245) | **Cancers** |
| **Chronic myeloid leukemia** | 0.00085 (0.00184) | 0.0097 (0.00227) | -0.00579 (0.0022) | **Cancers** |
| **Malignant skin melanoma** | -0.03193 (0.00188) | 0.04443 (0.00229) | -0.04711 (0.00219) | **Cancers** |
| **Non-melanoma skin cancer** | -0.01849 (0.0016) | 0.03033 (0.00195) | -0.03062 (0.00188) | **Cancers** |
| **Lip & oral cavity cancer** | -0.01237 (0.00181) | 0.01074 (0.00225) | -0.00149 (0.00218) | **Cancers** |
| **Brain & nervous system cancer** | -0.00952 (0.00187) | 0.02214 (0.00229) | -0.02003 (0.00222) | **Cancers** |
| **Breast cancer** | -0.00487 (0.00174) | 0.00384 (0.00215) | 0.00259 (0.00208) | **Cancers** |
| **Nasopharynx cancer** | -0.00339 (0.00313) | -0.0122 (0.00387) | 0.02611 (0.00372) | **Cancers** |
| **Acute lymphoid leukemia** | -0.00274 (0.00218) | 0.04947 (0.00255) | -0.04697 (0.00247) | **Cancers** |
| **Ovarian cancer** | -0.00161 (0.00157) | -0.00194 (0.00194) | 0.00084 (0.00188) | **Cancers** |
| **Prostate cancer** | -0.00083 (0.00168) | -0.01727 (0.00206) | 0.01778 (0.00199) | **Cancers** |
| **Aortic aneurysm** | 0.01846 (0.00185) | -0.00975 (0.00232) | 0.01184 (0.00224) | **Cardiovascular diseases** |
| **Ischemic heart disease** | 0.00817 (0.00198) | 0.01635 (0.00243) | -0.00731 (0.00237) | **Cardiovascular diseases** |
| **Cardiomyopathy & myocarditis** | 0.00501 (0.00164) | -0.00631 (0.00202) | 0.01314 (0.00194) | **Cardiovascular diseases** |
| **Peripheral vascular disease** | 0.00321 (0.00154) | -0.0067 (0.0019) | 0.01153 (0.00183) | **Cardiovascular diseases** |
| **Cardiovascular diseases** | 0.00314 (0.00207) | 0.01608 (0.00255) | -0.00566 (0.00248) | **Cardiovascular diseases** |
| **Rheumatic heart disease** | -0.02314 (0.0017) | 0.00801 (0.00216) | -0.0064 (0.00209) | **Cardiovascular diseases** |
| **Endocarditis** | -0.0103 (0.00153) | -0.00255 (0.00191) | 0.00108 (0.00185) | **Cardiovascular diseases** |
| **Atrial fibrillation & flutter** | -0.0098 (0.00149) | 0.00039 (0.00185) | -0.0059 (0.00179) | **Cardiovascular diseases** |
| **Cerebrovascular disease** | -0.00716 (0.0017) | 0.00945 (0.0021) | -0.00481 (0.00204) | **Cardiovascular diseases** |
| **Ischemic stroke** | -0.00678 (0.0016) | 0.00902 (0.00198) | -0.00657 (0.00191) | **Cardiovascular diseases** |
| **Hemorrhagic stroke** | -0.00642 (0.002) | 0.00822 (0.00248) | 0.00207 (0.0024) | **Cardiovascular diseases** |
| **Hypertensive heart disease** | -0.00593 (0.0016) | 0.00208 (0.00198) | 0.00132 (0.00191) | **Cardiovascular diseases** |
| **Other cardiovascular** | -0.00247 (0.00155) | -0.01157 (0.00191) | 0.01146 (0.00185) | **Cardiovascular diseases** |
| **% Screened** | 0.01576 (0.00171) | -0.02907 (0.00209) | 0.03201 (0.002) | **Clinical Care** |
| **% Vaccinated** | 0.00738 (0.0018) | -0.02111 (0.00221) | 0.02475 (0.00212) | **Clinical Care** |
| **Preventable Hosp. Rate** | 0.00646 (0.00187) | -0.00143 (0.00231) | 0.0095 (0.00223) | **Clinical Care** |
| **% With Access** | 0.00164 (0.00204) | -0.0122 (0.00251) | 0.00842 (0.00243) | **Clinical Care** |
| **Dentist Rate** | -7e-05 (0.00178) | -0.01495 (0.00217) | 0.0116 (0.0021) | **Clinical Care** |
| **PCP Rate** | -0.01285 (0.00184) | -0.01873 (0.00228) | 0.01706 (0.00221) | **Clinical Care** |
| **MHP Rate** | -0.00418 (0.00181) | -0.02169 (0.00219) | 0.01594 (0.00213) | **Clinical Care** |
| **Alcohol use disorders** | 0.00438 (0.00214) | -0.01422 (0.00263) | 0.0035 (0.00256) | **Deaths of Despair** |
| **Self-harm** | -0.0148 (0.00192) | 0.01795 (0.00237) | -0.02653 (0.00226) | **Deaths of Despair** |
| **Drug use disorders** | -0.00594 (0.00168) | 0.00559 (0.00208) | -0.00278 (0.00201) | **Deaths of Despair** |
| **Interpersonal violence** | -0.00434 (0.0021) | -0.01319 (0.00259) | 0.02011 (0.00249) | **Deaths of Despair** |
| **% Excessive Drinking** | 0.03145 (0.00177) | -0.02039 (0.00227) | 0.02113 (0.00219) | **Health Behaviors** |
| **% Smokers** | 0.02974 (0.0022) | -0.01839 (0.00278) | 0.03178 (0.00265) | **Health Behaviors** |
| **obesity_crude** | 0.01653 (0.00168) | -0.00315 (0.00212) | 0.00693 (0.00204) | **Health Behaviors** |
| **Food Environment Index** | 0.01521 (0.00207) | -0.0098 (0.00257) | 0.0099 (0.00249) | **Health Behaviors** |
| **% Insufficient Sleep** | 0.01186 (0.00187) | -0.01616 (0.00231) | 0.02684 (0.0022) | **Health Behaviors** |
| **Drug Overdose Mortality Rate** | 0.00779 (0.00198) | -0.01114 (0.00236) | 0.01197 (0.00224) | **Health Behaviors** |
| **physical_inactivity_crude** | 0.0076 (0.00189) | 0.02721 (0.0023) | -0.01822 (0.00225) | **Health Behaviors** |
| **diabetes_crude** | 0.0066 (0.00171) | 0.00217 (0.00212) | 0.00337 (0.00205) | **Health Behaviors** |
| **% Food Insecure** | -0.02113 (0.00218) | -0.00412 (0.00274) | 0.00892 (0.00264) | **Health Behaviors** |
| **opioid_prescribing_rate** | -0.01436 (0.0017) | 0.02187 (0.00206) | -0.01806 (0.00201) | **Health Behaviors** |
| **MV Mortality Rate** | -0.01329 (0.00226) | 0.03751 (0.00275) | -0.03658 (0.00265) | **Health Behaviors** |
| **Teen Birth Rate** | -0.00465 (0.0025) | 0.04352 (0.00298) | -0.03747 (0.00291) | **Health Behaviors** |
| **Age-Adjusted Mortality (White)** | 0.0086 (0.00253) | 0.01857 (0.00361) | -0.01345 (0.0035) | **Health Outcomes** |
| **Infant Mortality Rate** | 0.00699 (0.00262) | -0.00272 (0.00337) | 0.00692 (0.00328) | **Health Outcomes** |
| **YPLL Rate (White)** | 0.00568 (0.00238) | 0.01431 (0.00341) | -0.01074 (0.0033) | **Health Outcomes** |
| **Life Expectancy (Black)** | 0.00159 (0.00202) | -0.00679 (0.00296) | 0.00391 (0.00284) | **Health Outcomes** |
| **Age-Adjusted Mortality** | 0.00144 (0.0022) | 0.01559 (0.00268) | -0.0079 (0.00261) | **Health Outcomes** |
| **HIV Prevalence Rate** | -8e-05 (0.00192) | -0.01324 (0.00249) | 0.01813 (0.00237) | **Health Outcomes** |
| **Mentally Unhealthy Days** | -0.00882 (0.00202) | -0.01303 (0.00249) | 0.01712 (0.0024) | **Health Outcomes** |
| **Life Expectancy (White)** | -0.00766 (0.00249) | -0.0293 (0.00338) | 0.02353 (0.00331) | **Health Outcomes** |
| **% LBW** | -0.0075 (0.00183) | -0.00529 (0.00225) | 0.01323 (0.00216) | **Health Outcomes** |
| **% Frequent Mental Distress** | -0.00627 (0.00233) | -0.01594 (0.00287) | 0.02095 (0.00276) | **Health Outcomes** |
| **Child Mortality Rate** | -0.00508 (0.00231) | 0.00608 (0.00292) | -0.00487 (0.00281) | **Health Outcomes** |
| **Physically Unhealthy Days** | -0.00491 (0.00231) | -0.00734 (0.00286) | 0.01246 (0.00276) | **Health Outcomes** |
| **% Fair/Poor** | -0.00346 (0.00296) | -0.00126 (0.00366) | 0.01183 (0.00353) | **Health Outcomes** |
| **Life Expectancy** | -0.00343 (0.00211) | -0.01337 (0.00256) | 0.00682 (0.00249) | **Health Outcomes** |
| **% Frequent Physical Distress** | -0.00243 (0.00256) | -0.01004 (0.00316) | 0.01598 (0.00305) | **Health Outcomes** |
| **Age-Adjusted Mortality (Black)** | -0.00111 (0.00165) | 4e-04 (0.00247) | -0.00089 (0.00236) | **Health Outcomes** |
| **Years of Potential Life Lost Rate** | -0.00088 (0.00214) | 0.00946 (0.00264) | -0.00458 (0.00256) | **Health Outcomes** |
| **YPLL Rate (Black)** | -0.00044 (0.00167) | 9e-05 (0.00249) | -0.00041 (0.00239) | **Health Outcomes** |
| **Diarrheal diseases** | 0.01256 (0.00157) | -0.0225 (0.00192) | 0.02411 (0.00185) | **Infectious diseases** |
| **Meningitis** | -0.02166 (0.00242) | 0.00242 (0.00303) | 0.00725 (0.00293) | **Infectious diseases** |
| **Hepatitis** | -0.01431 (0.0016) | 0.02104 (0.00196) | -0.02228 (0.00189) | **Infectious diseases** |
| **Lower respiratory infections** | -0.00865 (0.00176) | 0.01075 (0.00218) | -0.00335 (0.00212) | **Infectious diseases** |
| **Tuberculosis** | -0.00658 (0.00287) | -0.01073 (0.00355) | 0.02286 (0.00342) | **Infectious diseases** |
| **HIV AIDS** | -0.00277 (0.00175) | -0.00239 (0.00216) | 0.00872 (0.00209) | **Infectious diseases** |
| **prcnt_bachelor_25_64_with_private_ins** | 1e-04 (0.00208) | 0.00361 (0.00258) | 0.00062 (0.00249) | **Insurance and Healthcare cost** |
| **prct_female_18_64_medicaid** | 0.04083 (0.00243) | -0.05852 (0.00296) | 0.05556 (0.00287) | **Insurance and Healthcare cost** |
| **prct_female_medicaid** | 0.03758 (0.00273) | -0.05347 (0.00334) | 0.05314 (0.00323) | **Insurance and Healthcare cost** |
| **prct_male_18_64_medicaid** | 0.03651 (0.00265) | -0.0609 (0.00319) | 0.05628 (0.00311) | **Insurance and Healthcare cost** |
| **prcnt_yes_highs_25_64_with_ins** | 0.0363 (0.00175) | -0.04004 (0.0022) | 0.0391 (0.00212) | **Insurance and Healthcare cost** |
| **prct_male_medicaid** | 0.03246 (0.00288) | -0.0549 (0.0035) | 0.0536 (0.00338) | **Insurance and Healthcare cost** |
| **prcnt_no_highs_25_64_with_public_ins** | 0.02879 (0.00184) | -0.04725 (0.0022) | 0.04833 (0.00211) | **Insurance and Healthcare cost** |
| **prcnt_yes_highs_25_64_with_public_ins** | 0.02856 (0.0024) | -0.05231 (0.00288) | 0.05022 (0.00279) | **Insurance and Healthcare cost** |
| **prcnt_no_highs_25_64_with_ins** | 0.02625 (0.00158) | -0.03267 (0.00196) | 0.03222 (0.00189) | **Insurance and Healthcare cost** |
| **Percent Eligible for Medicaid** | 0.02606 (0.002) | -0.04563 (0.0024) | 0.04973 (0.00228) | **Insurance and Healthcare cost** |
| **prcnt_yes_highs_25_64_with_private_ins** | 0.0202 (0.00243) | -0.00435 (0.00303) | 0.00569 (0.00293) | **Insurance and Healthcare cost** |
| **prcnt_bachelor_25_64_with_public_ins** | 0.0185 (0.00216) | -0.02046 (0.00268) | 0.01699 (0.0026) | **Insurance and Healthcare cost** |
| **prct_male_under_18_medicaid** | 0.01216 (0.00253) | -0.02729 (0.0031) | 0.03038 (0.00299) | **Insurance and Healthcare cost** |
| **prct_female_under_18_medicaid** | 0.012 (0.00251) | -0.02448 (0.00308) | 0.02773 (0.00297) | **Insurance and Healthcare cost** |
| **prcnt_bachelor_25_64_with_ins** | 0.0116 (0.00167) | -0.0132 (0.00207) | 0.01461 (0.002) | **Insurance and Healthcare cost** |
| **prct_female_over_64_medicaid** | 0.01135 (0.00256) | -0.02814 (0.00314) | 0.03426 (0.00301) | **Insurance and Healthcare cost** |
| **prct_male_over_64_medicaid** | 0.00922 (0.00261) | -0.0253 (0.00321) | 0.02884 (0.00309) | **Insurance and Healthcare cost** |
| **w_prcnt_18_dis** | 0.00399 (0.00163) | -0.00353 (0.00202) | 0.00475 (0.00195) | **Insurance and Healthcare cost** |
| **prcnt_no_highs_25_64_with_private_ins** | 0.00314 (0.00191) | 0.00802 (0.00236) | -0.0095 (0.00228) | **Insurance and Healthcare cost** |
| **wnh_prcnt_18_dis** | 0.00257 (0.00187) | 0.00063 (0.00231) | 0.00106 (0.00224) | **Insurance and Healthcare cost** |
| **Percent Female** | -2.36267 (8.22311) | 29.51561 (10.15818) | -27.18413 (9.82706) | **Insurance and Healthcare cost** |
| **Part B Drugs Actual Costs** | -0.0654 (0.03587) | -0.04542 (0.04444) | 0.07531 (0.04298) | **Insurance and Healthcare cost** |
| **Uninsured %: All Incomes** | -0.03854 (0.00184) | 0.06718 (0.00211) | -0.06429 (0.00205) | **Insurance and Healthcare cost** |
| **Uninsured %: <= 138% of Poverty** | -0.03775 (0.00157) | 0.06141 (0.0018) | -0.05695 (0.00177) | **Insurance and Healthcare cost** |
| **Uninsured %: <= 400% of Poverty** | -0.03754 (0.00169) | 0.06359 (0.00194) | -0.05976 (0.0019) | **Insurance and Healthcare cost** |
| **Procedures Per Capita Actual Costs** | -0.02524 (0.00185) | 0.03314 (0.00228) | -0.02773 (0.00223) | **Insurance and Healthcare cost** |
| **Hospice Per Capita Actual Costs** | -0.01628 (0.00153) | 0.01399 (0.0019) | -0.01211 (0.00185) | **Insurance and Healthcare cost** |
| **Tests Per Capita Actual Costs** | -0.01566 (0.00169) | 0.02702 (0.00206) | -0.01681 (0.00203) | **Insurance and Healthcare cost** |
| **Emergency Department Visits** | -0.01558 (0.04555) | -0.26979 (0.05613) | 0.31343 (0.0542) | **Insurance and Healthcare cost** |
| **wnh_prcnt_65_dis** | -0.00934 (0.00231) | 0.03557 (0.00279) | -0.03238 (0.00271) | **Insurance and Healthcare cost** |
| **w_prcnt_65_dis** | -0.00719 (0.00199) | 0.02858 (0.00241) | -0.02642 (0.00234) | **Insurance and Healthcare cost** |
| **Imaging Per Capita Actual Costs** | -0.00717 (0.00179) | 0.02492 (0.00218) | -0.01672 (0.00213) | **Insurance and Healthcare cost** |
| **wnh_prcnt_18_64_dis** | -0.00405 (0.00265) | 0.01323 (0.00328) | -0.00839 (0.00317) | **Insurance and Healthcare cost** |
| **w_prcnt_18_64_dis** | -0.00129 (0.00206) | 0.00522 (0.00255) | -0.00092 (0.00246) | **Insurance and Healthcare cost** |
| **Mortality risk, age 65-85** | 0.00103 (0.00212) | 0.02114 (0.0026) | -0.00996 (0.00253) | **Life expectancy and Mortality** |
| **Mortality risk, age 0-5** | -0.02136 (0.00227) | 0.0251 (0.00281) | -0.01421 (0.00274) | **Life expectancy and Mortality** |
| **Mortality risk, age 5-25** | -0.01489 (0.00225) | 0.03097 (0.00274) | -0.02393 (0.00267) | **Life expectancy and Mortality** |
| **Mortality risk, age 25-45** | -0.01351 (0.00227) | 0.02252 (0.00279) | -0.01366 (0.00272) | **Life expectancy and Mortality** |
| **Mortality risk, age 45-65** | -0.004 (0.0023) | 0.02253 (0.00282) | -0.00938 (0.00275) | **Life expectancy and Mortality** |
| **Interstitial lung disease** | 0.00352 (0.00158) | -0.01676 (0.00193) | 0.01473 (0.00187) | **Respiratory diseases** |
| **Asthma** | 0.00101 (0.00184) | -0.01102 (0.00226) | 0.01022 (0.00219) | **Respiratory diseases** |
| **Other pneumoconiosis** | -0.02678 (0.0017) | 0.01137 (0.00218) | -0.00607 (0.00211) | **Respiratory diseases** |
| **Other chronic respiratory** | -0.01735 (0.00189) | 0.00058 (0.00237) | -0.00042 (0.00229) | **Respiratory diseases** |
| **Asbestosis** | -0.00749 (0.00142) | 0.0042 (0.00177) | -0.00312 (0.00171) | **Respiratory diseases** |
| **Pneumoconiosis** | -0.00608 (0.00148) | 0.00295 (0.00183) | -0.00066 (0.00177) | **Respiratory diseases** |
| **Chronic respiratory diseases** | -0.00548 (0.0019) | 0.03972 (0.00224) | -0.03285 (0.00219) | **Respiratory diseases** |
| **Chronic obstructive pulmonary** | -0.00506 (0.0019) | 0.04282 (0.00221) | -0.03577 (0.00218) | **Respiratory diseases** |
| **Silicosis** | -0.00484 (0.00144) | -0.00237 (0.00178) | -0.00024 (0.00173) | **Respiratory diseases** |
| **Coal workers pneumoconiosis** | -0.00282 (0.00147) | 0.00167 (0.00181) | 0.00044 (0.00175) | **Respiratory diseases** |
| **% Single-Parent Households** | 0.02735 (0.00218) | -0.04358 (0.00265) | 0.04947 (0.00252) | **Social, Physical and Economic Environment** |
| **% Homeowners** | 0.00679 (0.00233) | 0.00601 (0.00289) | -0.00108 (0.0028) | **Social, Physical and Economic Environment** |
| **Firearm Fatalities Rate** | -0.01567 (0.00207) | 0.02076 (0.0026) | -0.01949 (0.00251) | **Social, Physical and Economic Environment** |
| **% Severe Housing Problems** | -0.00761 (0.00331) | -0.01702 (0.00409) | 0.00787 (0.00396) | **Social, Physical and Economic Environment** |
| **% Disconnected Youth** | -0.00581 (0.00274) | 0.00273 (0.00349) | -0.00153 (0.00335) | **Social, Physical and Economic Environment** |
| **Injury Death Rate** | -0.00538 (0.00192) | 0.00969 (0.00236) | -0.01263 (0.00228) | **Social, Physical and Economic Environment** |
| **Violent Crime Rate** | -0.00248 (0.00181) | -0.003 (0.00223) | 0.00553 (0.00215) | **Social, Physical and Economic Environment** |
| **% Children in Poverty** | -0.00015 (0.00322) | -0.01096 (0.00398) | 0.02252 (0.00383) | **Social, Physical and Economic Environment** |
