# Supplementary figures and images for: Viewing the US presidential electoral map through the lens of public health
Source: PLoS One. 2021 Jul 21;16(7):e0254001. doi: 10.1371/journal.pone.0254001 (PMC8294501; doi:10.1371/journal.pone.0254001)

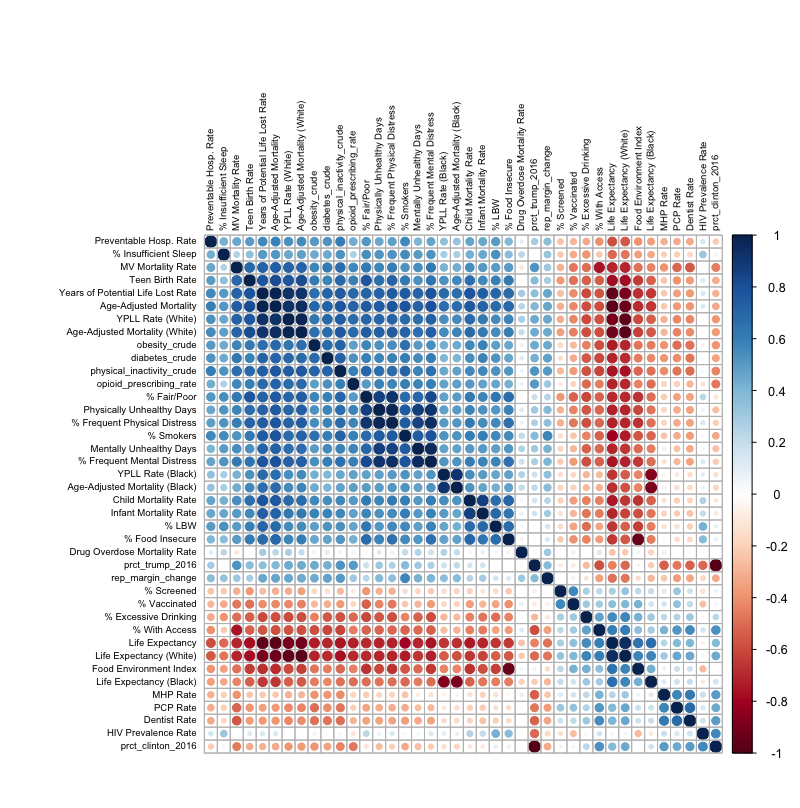

Supplement: S2 Fig — (TIFF) [file pone.0254001.s006.tiff]
