# Supplementary material for: Viewing the US presidential electoral map through the lens of public health
Source: PLoS One. 2021 Jul 21;16(7):e0254001. doi: 10.1371/journal.pone.0254001 (PMC8294501; doi:10.1371/journal.pone.0254001)
Supplement: Attachment — Submitted filename: D. Storms - Review of Revised Voting Patterns and Health, Wellness, Mortality .docx [file pone.0254001.s009.docx]

Dory Storms Review of PONE-D-20-17485R1 [EM1D 5fysbb930863729]

**Title: Viewing the US presidential electoral map through the lens of public health-Rev 1**

The authors have greatly improved this draft paper by adding a literature review of peer-reviewed sources that places this research within the context of other published studies on voting patterns and health, well-being and disease conditions. The authors now, at the beginning of the paper, define key terms and use them consistently in the text. The meaning of the partisan categorization of counties, districts, and states cited in this paper is now clear, as is the reason for the study’s use of variables at the county level.

In addition to clarifying terms and adding a literature review, the authors review findings in recent studies on the socio-economic and demographic differences found between Republican and Democratic counties. They describe these differences found in characteristics at one point in time, but also in changes over time in economic fortunes and wealth, (e.g. trends in median household income). This is especially important because as the authors note, these socio economic and demographic characteristics are drivers of health care differences, and as their analyses show, of political voting patterns.

Further, the authors add information on recent studies regarding the party-specific priorities in healthcare costs and policy issues such as Medicare reform and the ACA. Another welcome change in the manuscript is the thorough discussion of this study’s limitations.

In summary, the responsiveness of the authors to reviewers’ suggestions, and their subsequent hard work in adding multivariate analyses, has made this a stronger and more competitive paper. However, there is further to go.

The main question, the whole rationale of this paper is too vague. What do the authors hope to accomplish? What difference might the findings make in real life? In the Abstract, the authors say “it is important to understand the relationship between voting “patterns, health, disease, and mortality.” Why? How do you measure “understand”? Will the reader understand” the relationship any better after at least 20 straight pages of minimal text “data dump”? Doubtful.

How much of the associations between voting patterns, health, disease and mortality can be explained by social economic and demographic factors? According to the authors, the multivariate analyses showed the socio demographic factors were the controlling factors, wiping out the need to include in the main body of the text, approximately 20 pages listing the individual associations found between voting patterns and 150 public health variables, in four slightly different forms of statistical analyses. The authors state that they wanted the paper to be a bit of a data dump because it might be a resource for people and help generate ideas. That is a little like dragging a large clothes rack behind one, hoping to generate ideas from others about good clothing combinations to wear that the owner was unable, or did not, identify that morning. Why not put these endless tables in the appendices? They can be a resource there, too. However, the authors should keep within the text a description of the main findings of Tables 1-4 plus supplementary tables, and the very informative Figures, and their discussion should remain in the text, as well as a full discussion of the main findings of the multivariate analyses.

The Introduction actually begins with a reasonable way to bring together the various strands into one coherent argument: healthcare costs and policy as a top priority for the public, and voters, (and for the incoming administration). The authors need to focus the purpose of this paper more on this concrete issue of concern to people, instead of reference to some vague good- for-you better understanding of relationships between voting patterns and a variety of public health variables. The authors already have all the necessary elements to accomplish this: the literature review; the interesting findings about insurance and voting patterns, and trends in both; the discussion of access rural/urban differences, the discussion around results pertaining to Medicare, Medicaid, ACA, expansion of health care reform, and the overall conclusion that counties going for the Republican candidate have worse health outcomes, and thus presumably the implied need for more targeted health care policies, increased access, and affordable health insurance to offset costs of care to manage their worse health status and poorer life chances.

Since voting patterns are associated with health behaviors and health outcomes, the results of this study might indicate, for example, need for funding of special health education initiatives for rural, older, economically marginal citizens set in Republican counties, a departure from rewarding voters for the winning political party, but essential action if we are to achieve equitable health care in this country. Public health must maintain a bridge between “understanding” and public action. Marshalling the paper’s focus and prioritization on healthcare policy makes it understandable why the reader should care about this paper and how it can be instrumental in answering current day concerns of ordinary people as well as a valuable source document for policy initiators and legislators.

A final word about the power of socio-economic and characteristics on disease, well-being, preferences and behaviors. We tend to look at those characteristics as reflective of the individual, but they are also reflective of the culture and thus society’s view of the caste of the individual within the culture. Much has been said recently, for very good reasons, about America’s racism. Unfortunately, America is also ageist; youth is highly valued, but older people in general are not considered of value to the economy, culture, governance, nor creation of new ideas, and are often regarded as intellectually less capable, and less physically appealing. Rural areas in America are considered to reflect the past while urban areas are considered not only the future, but also the current’ ‘place where things are happening’. Wealth is admired in the USA, envied, and wealthy people even fawned over, while the poor are considered to have some personal flaw, perhaps lazy or too dumb, and of little value to the community -- possibly even a threat to others, needing surveillance. College is regarded as essential for any good paying job, and stopping at high school or dropping out is a sign of failure. Since Republican counties have more households than Democratic counties categorized as rural, white, older, of lower household income, and fewer with any college, then the only culturally redeeming socio-demographic characteristic left in Republican counties is being white. Any wonder White Supremacy has made inroads in such counties?

To be judged the lowest of the low by the larger society lays a substantial emotional burden on personal well-being, as tough as lack of access to quality and quantity of healthcare, or inability to pay for healthcare costs. We have begun to acknowledge this assessment and our role in maintaining such cultural judgements when we are forced to address the health care disparities afflicting Black and brown urban residents. Extending that painful learning to our view of older, poor, rural, less educated residents of counties that voted Republican in the 2016 election, will take us a step closer to emotionally understanding the relationship” between their health measures and their voting patterns. When the culture has such a damning view of the social and demographic characteristics of an aging individual, with increasing negative health outcomes and decreased life expectancy, one can understand the appeal of the amended slogan: Make Me, this American, Great Again. Despite how puzzling it may seem to outsiders when an individual with poor health outcomes does not vote for personally advantageous healthcare reform but for a self-betterment slogan instead, it is not so clear that he/she may be voting against their own self-interest.

You have a fine, important paper. Now bring it all together, tighten it-- purpose, priority, recommendations based on results and discussion.
