# Supplementary material for: Viewing the US presidential electoral map through the lens of public health
Source: PLoS One. 2021 Jul 21;16(7):e0254001. doi: 10.1371/journal.pone.0254001 (PMC8294501; doi:10.1371/journal.pone.0254001)
Supplement: Attachment — Submitted filename: TymorH-health-disp-coverLetter-rev2.docx [file pone.0254001.s010.docx]

**May 7, 2021**

We would like to thank the editor for closely reading this manuscript as well as the reviewer comments and our responses to them. In addition to addressing reviewer comments we have streamlined the overall text and links to the supplemental material. We detail our full response to the review comments below.

**Reviewer 1:**

We want to thank the reviewer for all of their helpful, productive comments in this process.

We have addressed reviewer #1’s point to include the variance explained of the first 2 principle components, as well as noting the variables that are associated with those principle components. We have also removed the supplementary tables from the manuscript, and into the appendix.

**Reviewer 2:**

We want to thank the reviewer for their expertise in guiding the process of refining this paper and we appreciate the reviewer’s kind words.

We appreciate their comment regarding the clarity of the paper and have taken steps to improve the general understanding of the goal of our work.

We agree that the majority of tables should be included as an appendix and have modified the manuscript so they are not in the body of the manuscript.

We particularly appreciate the reviewer’s specific comments regarding how to make this work applicable beyond the realm of pure academic interest. We are similarly interested in how this sort of work can have actionable consequences. As such, we have modified the manuscript in particular sections to reflect our belief that these data should guide policy.

**Thank you for your consideration.**

Sincerely,

Rich, Tymor, Jonathan and Michael,

Dr. Richard Bonneau,

director NYU Center for Genomics and Systems Biology

Professor, New York University

Joint in Depts. of Biology and Computer Science

Group Leader, Systems Biology,

CCB Simons Foundation

[bonneau@nyu.edu](mailto:bonneau@nyu.edu)

voice: (01) 917-678-1102
